# Analyzing Comorbidity Patterns in Patients With Thyroid Disease Using Large-Scale Electronic Medical Records: Network-Based Retrospective Observational Study

**DOI:** 10.2196/54891

**Published:** 2024-10-03

**Authors:** Yanqun Huang, Siyuan Chen, Yongfeng Wang, Xiaohong Ou, Huanhuan Yan, Xin Gan, Zhixiao Wei

**Affiliations:** 1 Department of Medical Equipment The First Affiliated Hospital of Guangxi Medical University Nanning, Guangxi China; 2 Department of Nuclear Medicine The First Affiliated Hospital of Guangxi Medical University Nanning, Guangxi China

**Keywords:** thyroid disease, comorbidity patterns, prevalence, network analysis, electronic medical records

## Abstract

**Background:**

Thyroid disease (TD) is a prominent endocrine disorder that raises global health concerns; however, its comorbidity patterns remain unclear.

**Objective:**

This study aims to apply a network-based method to comprehensively analyze the comorbidity patterns of TD using large-scale real-world health data.

**Methods:**

In this retrospective observational study, we extracted the comorbidities of adult patients with TD from both private and public data sets. All comorbidities were identified using ICD-10 (International Classification of Diseases, 10th Revision) codes at the 3-digit level, and those with a prevalence greater than 2% were analyzed. Patients were categorized into several subgroups based on sex, age, and disease type. A phenotypic comorbidity network (PCN) was constructed, where comorbidities served as nodes and their significant correlations were represented as edges, encompassing all patients with TD and various subgroups. The associations and differences in comorbidities within the PCN of each subgroup were analyzed and compared. The PageRank algorithm was used to identify key comorbidities.

**Results:**

The final cohorts included 18,311 and 50,242 patients with TD in the private and public data sets, respectively. Patients with TD demonstrated complex comorbidity patterns, with coexistence relationships differing by sex, age, and type of TD. The number of comorbidities increased with age. The most prevalent TDs were nontoxic goiter, hypothyroidism, hyperthyroidism, and thyroid cancer, while hypertension, diabetes, and lipoprotein metabolism disorders had the highest prevalence and PageRank values among comorbidities. Males and patients with benign TD exhibited a greater number of comorbidities, increased disease diversity, and stronger comorbidity associations compared with females and patients with thyroid cancer.

**Conclusions:**

Patients with TD exhibited complex comorbidity patterns, particularly with cardiocerebrovascular diseases and diabetes. The associations among comorbidities varied across different TD subgroups. This study aims to enhance the understanding of comorbidity patterns in patients with TD and improve the integrated management of these individuals.

## Introduction

Thyroid disease (TD) is a prominent endocrine disorder and has become an increasing public health concern worldwide. In the United States, its prevalence rose from 2.54% in 1999-2002 to 5.05% in 2015-2018 [1]. Thyroid cancer (TC) is considered the 9th most common malignancy and the most common endocrine cancer, accounting for over 586,000 cases and 43,600 deaths annually [2]. In China, thyroid nodules (TNs) were the most common form of TD, affecting about 36.9% of the population in 2017 [3]. The incidence of TC in China is nearly 2 times the global average, with 11.3 versus 6.6 cases per 100,000 people [4].

TD is intricately linked to multiple diseases and health conditions [5,6]. A comprehensive understanding of the interactions and overlapping symptoms among these diseases can enhance clinicians’ diagnostic accuracy and personalize treatment plans [7]. However, previous studies have mostly focused on the correlation between a single disease and a specific type of TD [6,8], neglecting the simultaneous consideration of multiple diseases. Consequently, the underlying patterns of TD’s multiple comorbidities are far from fully elucidated.

Advancements in network theory offer fresh insights into understanding the intricate relationships among comorbidities. Recently, the phenotypic comorbidity network (PCN) has gained popularity in exploring associations and disparities across multiple diseases. In the PCN, coexisting diseases are represented as nodes, with edges indicating their connections. These edges can be assigned weights to reflect the frequency of their coexistence. The PCN uncovers hidden disease patterns, facilitating enhanced comorbidity risk assessments and future disease predictions for individuals. Recently, researchers have used the PCN to reveal comorbidity patterns for specific diseases such as diabetes [9], colorectal cancer [10], and heart failure [11].

Electronic medical records (EMRs), a typical real-world data set, are increasingly mined for valuable insights enriched with clinical history information to enhance decision-making [12]. As a crucial component of EMR, discharge diagnoses reflect patients’ co-occurring diseases and health status during hospitalization, offering insights into comorbidity associations. These disease diagnoses are typically identified by standardized International Classification of Diseases (ICD) codes, which have proven effective in elucidating comorbidity patterns among diseases in prior studies [7,10,13].

In this study, we aimed to leverage a network-based method to systematically investigate comorbidity patterns in a general population of patients with TD, using disease codes from large-scale real-world EMR data from both private and public data sets. We hope to uncover previously recognized or unrecognized relationships among multiple comorbidities of TD.

## Methods

### Study Populations

**Figure 1:**
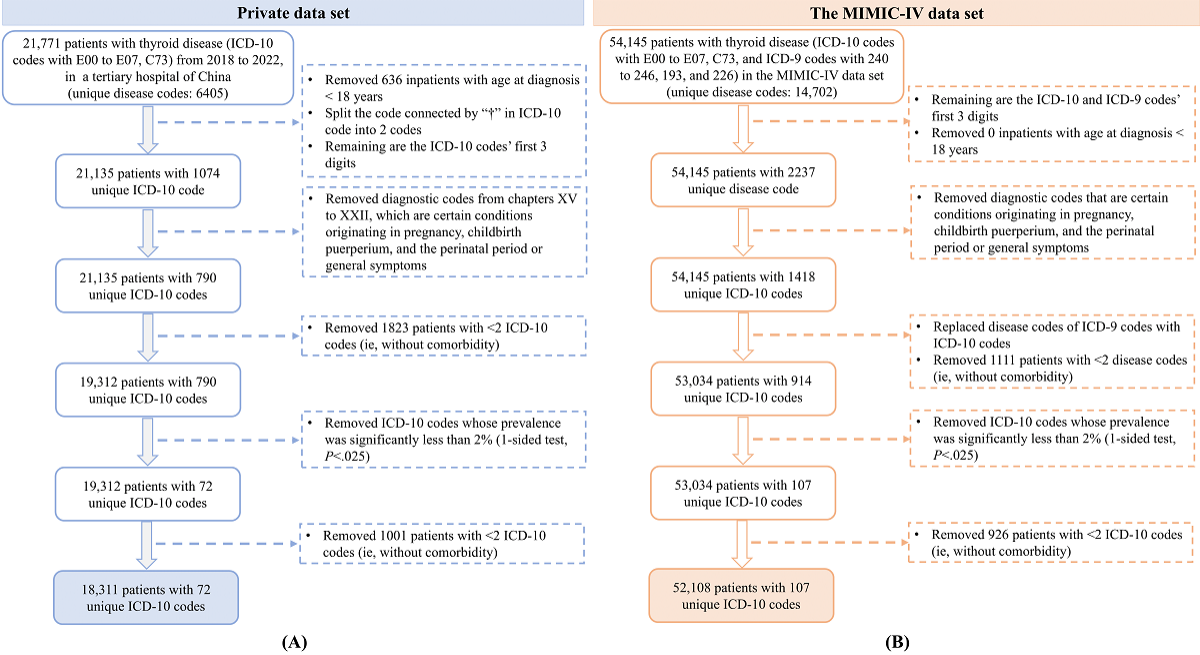
Flowchart of the study participants. ICD-10: International Classification of Diseases, 10th Revision.

We conducted a retrospective cohort study using EMR data from both private and public data sets. Age, sex, and ICD codes were extracted. The private data set, covering 2018-2022, was obtained from a tertiary hospital in Guangxi, China, and included 21,771 hospitalizations and 6405 distinct ICD-10 (10th Revision) codes. Patients with TD were identified by ICD-10 codes E00-E07 (benign TD [BTD]) or C73 (TC). We treated each hospitalization record as an independent individual, considering that patients may have different health states and disease progression during different hospitalizations. The data cleaning process to identify the study cohort was as follows: (1) included adult patients aged ≥18 years; (2) excluded diagnostic codes from chapters XV to XXII, which pertain to pregnancy, childbirth, puerperium, perinatal conditions, and general symptoms; (3) excluded patient records with fewer than 2 ICD-10 codes; and (4) excluded rare diseases with a prevalence <2%. Ultimately, the study population comprised 18,311 patients in the private data set (Figure 1A).

Additionally, we validated our study using the Medical Information Mart for Intensive Care (MIMIC)-IV data set, a public clinical database containing over 73,000 admissions from 2008 to 2019. Diseases were initially coded using ICD-10 and ICD-9 (9th Revision) codes. To ensure consistency, we standardized all disease codes to 3-digit ICD-10 codes. Ultimately, we extracted data for 52,108 patients with TD from the MIMIC-IV data set (Figure 1B).

### Ethical Considerations

The original data collection for this study was approved by the Medical Ethics Committee of the First Affiliated Hospital of Guangxi Medical University (approval number 2023-E592-01), and the Ethics Committee waived the requirement for a consent form. Informed consent from patients to use their data could not be obtained because the study is retrospective, observational, and population based. The analysis of the information was anonymized, with no variables that could identify individual patients. Additionally, we collected public clinical data from the MIMIC database after completing the web course (certification number 57439457). Given that the MIMIC-IV database consists of deidentified data and is publicly accessible, and as the collection of patient information and the creation of the research resource were reviewed by the Institutional Review Board at Beth Israel Deaconess Medical Center, which granted a waiver of informed consent and approved the data-sharing initiative [14], this study was not subject to specific ethical review.

### Comorbidity Prevalence

We stratified the population by sex (male or female) and disease type (TC or BTD). The prevalence of all diseases with ICD-10 codes and the 95% CI were calculated. We excluded diseases with a prevalence of <2% (*P*<.03, 1-sided test) in any subgroup to avoid including rare diseases. The lists of 72 and 107 diseases from the private and MIMIC-IV data sets are presented in Tables S1 and S2 in Multimedia Appendix 1, respectively. TDs, including TC (C73), nontoxic goiter (E04), hypothyroidism (E02 and E03), hyperthyroidism (E05), and other BTDs (E06), were excluded from the list of diseases, resulting in a final count of 67 and 102 comorbidities in the private and MIMIC-IV data sets, respectively. After comparing the comorbidity differences between the TC and BTD subgroups, we analyzed the comorbidity differences between patients with TC and those with specific typical BTDs (nontoxic goiter, E04; hypothyroidism, E02 and E03; and hyperthyroidism, E05). Subgroup details are presented in Figure S1 in Multimedia Appendix 1.

To evaluate the differences in comorbidity prevalence by sex and disease type, we calculated relative differences and used a *Z*-test for significance. The calculation for the relative difference by sex is based on equation (1) [10]:

d = (P_male_ – P_female_)/[(P_male_ + P_female_)/2] **(1)**

where *P*_male_ and *P*_female_ represent the prevalences of comorbidities in males and females, respectively.

We considered comorbidity prevalence to be significantly different if the relative difference exceeded 0.1 [10] and the absolute difference was statistically significant. Among these, comorbidities with a ≥0.5-fold prevalence increase were deemed enriched. We used Spearman correlation to assess comorbidity prevalence trends with age and applied K-means clustering to group comorbidities by age-related prevalence.

### Phenotypic Comorbidity Network Construction

We constructed a PCN to capture the coexistence of multiple diseases. In the PCN, nodes represent disease codes (ICD-10 codes at 3 digits) that are connected by edges. Node sizes are proportional to disease prevalence, and node colors represent the ICD-10 categories. The cosine index was applied to quantify the comorbidity strength of coexisting diseases, taking into account the co-occurrence and prevalence of comorbidities, thereby minimizing the influence of sample size. We defined a cutoff value to detect comorbidity coexistence measured by the cosine index by assessing the relationship between the Pearson correlation and cosine index, where the number of significant coexisting comorbidities was equal in both networks as measured by the cosine index and Pearson correlation. The cosine index and Pearson correlation coefficient are defined in equations (2) and (3), respectively, and the significance of 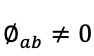 was determined by performing a *t* test (2-tailed), calculated according to equation (4) [10].



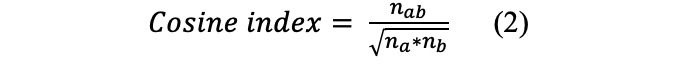





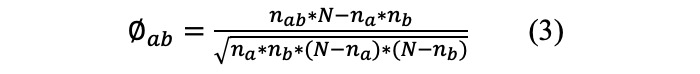





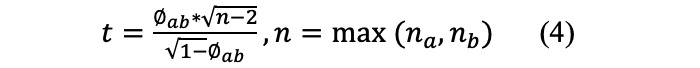



where *N* is the total number of patients with TD; and *n_a_*, *n_b_*, and *n_ab_* are the number of patients with disease a, disease b, and both diseases, respectively.

Four structural properties of the PCN were measured using network indices, including network density, degree, average degree of neighbors, and betweenness centrality [15]. Network density measures the compactness of a network by calculating the ratio of significant connections to all potential ones. A denser network indicates more connections among comorbidities. The degree of the PCN reflects its number of connections with other comorbidities. When a comorbidity is directly connected to others, those are referred to as neighbors. We calculated the average degree of neighbors to measure neighbor connectivity. Betweenness centrality represents the number of shortest paths between any 2 comorbidities. A high betweenness centrality indicates a greater likelihood of forming bridges between other comorbidities or serving as endpoints for many comorbidities [10].

To identify the most important comorbidity in the PCN, we applied the PageRank algorithm, which considers edge weights [16]. The PageRank algorithm calculates the importance of each node in a network by assigning ranks. Nodes that are connected to other nodes with a high rank receive a higher weight and are considered more central, while a higher PageRank value for a node indicates greater influence within the network. Comorbidities with the top 5 PageRank values were defined as the most important comorbidities in the PCN.

We constructed 4 separate PCNs for males, females, patients with TC, and patients with BTD. We then compared comorbidity strengths to measure disparities by sex and disease type. When a coexisting disease was unique or enriched (with a comorbidity strength of at least 0.05 higher than that of another) in a given subgroup, the difference was considered significant and was defined as an abundant connection [10].

Analyses and visualizations were performed using R 4.3.1 (R Foundation) and Python 3.7 (Python Foundation), leveraging the igraph and ggraph libraries for network visualization and property computation in R, and the pyecharts package for generating Sankey diagrams in Python.

## Results

### Characteristics of Patients With Thyroid Disease

In the private data set, the median age of patients with TD was 58 years, with 61.09% (11,187/18,311) being female and 6.25% (1144/18,311) having TC (Table 1). By contrast, in the MIMIC-IV data set, patients with TD were older, with a median age of 67 years; among them, 71.74% (37,384/52,108) were female and less than 0.93% (482/52,108) had TC. Patients with TD exhibited a high comorbidity burden, with a median number of comorbidities of 5 in the private data set and 7 in the MIMIC-IV data set. Males had more comorbidities than females, with the median number of comorbidities significantly higher in males than in females (6 vs 4, *P*<.001, and 8 vs 7, *P*<.001, in the private and MIMIC-IV data sets, respectively). Patients with TC were younger and had fewer comorbidities than patients with BTD. The median age of patients with TC was 16 and 11 years younger than that of patients with BTD, and the median number of comorbidities in patients with TC was 2 and 3 fewer than that in patients with BTD, in the private and MIMIC-IV data sets, respectively.

**Table 1:** Characteristics of patients with thyroid disease on the 2 data sets.

Demographic and clinical factors	Private data set						MIMIC^a^-IV data set				
	Overall	Male	Female	TC^b^	BTD^c^	Overall		Male	Female	TC	BTD
Number of patients	18,311	7124	11,187	1144	17,167	52,108		14,724	37,384	482	51,626
Age (years), median (IQR)	58 (49-67)	59 (51-68)	57 (47-66)	43 (34-53)	59 (50-67)	67 (55-78)		68 (57-78)	67 (55-79)	56 (45-70)	67 (55-79)
Number of comorbidities, median (IQR)	5 (3-7)	6 (4-8)	4 (3-6)	3 (2-4)	5 (3-7)	7 (5-10)		8 (5-11)	7 (5-10)	4 (3-6)	7 (5-10)

^a^MIMIC: Medical Information Mart for Intensive Care.

^b^TC: thyroid cancer.

^c^BTD: benign thyroid disease.

**Figure 2:**
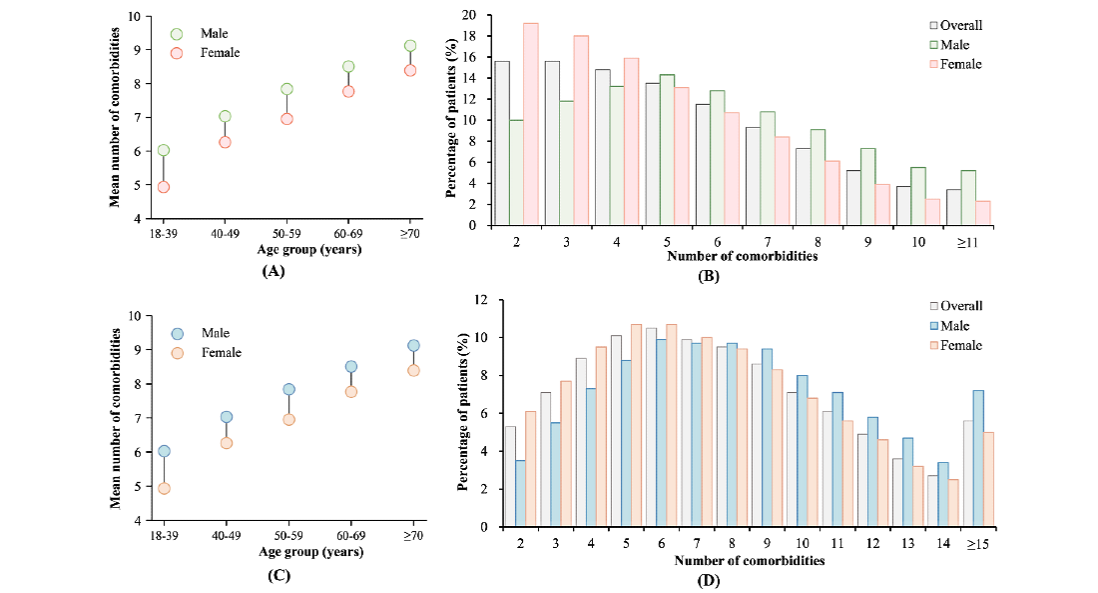
Comorbidity and age distributions in different subgroups of patients with thyroid disease. (A) Age-specific mean number of comorbidities and (B) frequency of patients per distinct number of comorbidities per patient on the private data set, respectively. Panels (C) and (D) are the counterparts of (A) and (B) on the MIMIC-IV data set, respectively. MIMIC: Medical Information Mart for Intensive Care.

The mean number of comorbidities increased with age in both males and females across both data sets (Figure 2A and 2C). In the private data set, the percentage of patients with TD decreased as the number of comorbidities increased, both overall and among females. By contrast, in the MIMIC-IV data set, the percentage of patients exhibited a trend of initially increasing and then decreasing with the number of comorbidities (Figure 2B and 2D).

**Figure 3:**
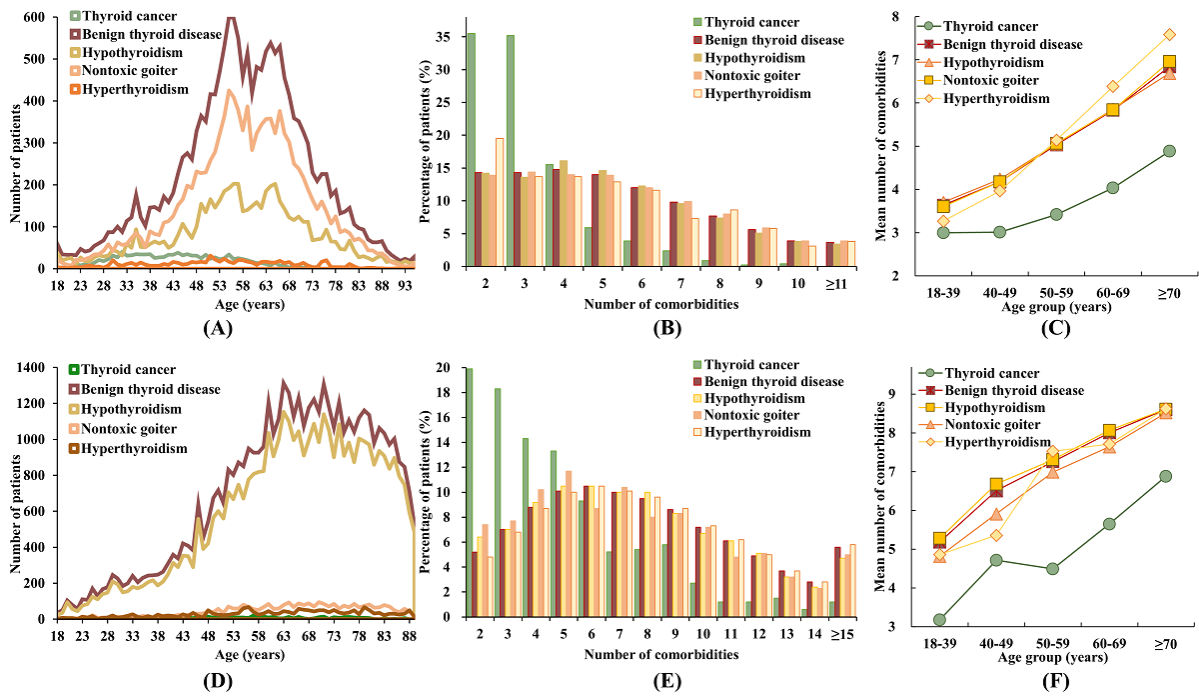
(A-C) The number of patients across different age groups, the percentage of patients with varying numbers of comorbidities, and the mean number of comorbidities for each thyroid disease subgroup, respectively, based on the private data set. (D-F) The corresponding data for patients from the MIMIC-IV data set. MIMIC: Medical Information Mart for Intensive Care.

The incidence of patients with different types of TDs varied between the 2 data sets (Figure 3). In the private data set, nontoxic goiter was the most common BTD, affecting 67.77% (12,410/18,311) of patients, whereas in the MIMIC-IV data set, hypothyroidism was prevalent in 86.36% (44,999/52,108) of patients. In most patient subgroups, the percentage of patients decreased with the number of comorbidities in the private data set, while in the MIMIC-IV data set, the percentage of patients initially rose and then declined as the number of comorbidities increased (Figure 3B and 3E). However, across all TD subgroups within both data sets, the mean number of comorbidities increased with age, with Spearman correlations of 0.993 (*P*=.001) and 0.955 (*P*<.001) in the private and MIMIC-IV data sets, respectively (Figure 3C and 3F).

### Comorbidity Prevalence and Differences Between Subgroups

**Figure 4:**
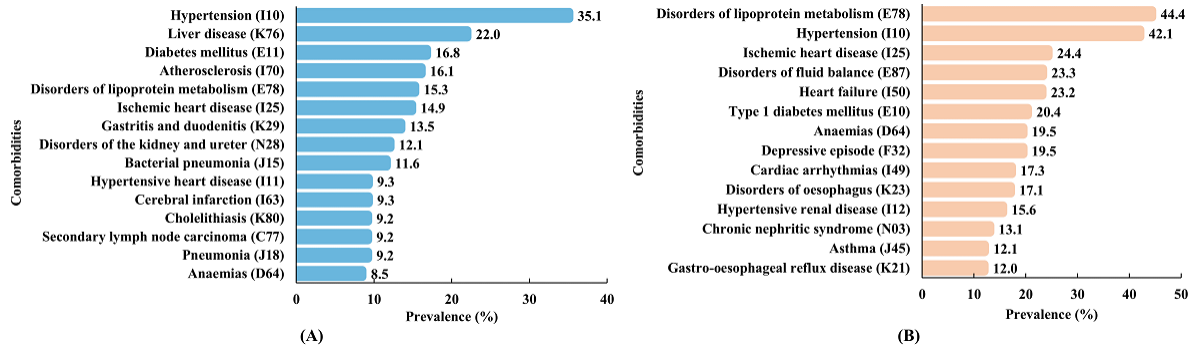
The top 15 comorbidities with the highest prevalence of patients with thyroid disease on the (A) private and (B) MIMIC-IV data sets. MIMIC: Medical Information Mart for Intensive Care.

Figure 4 displays the top 15 diseases with the highest prevalence. In the private data set, the most prevalent comorbidities were hypertension, liver disease, and diabetes, with prevalences of 35.14% (6435/18,311), 21.97% (4023/18,311), and 16.82% (3080/18,311), respectively. In the MIMIC-IV data set, hypertension and diabetes were also among the most common comorbidities, with prevalence rates of 42.13% (21,951/52,108) and 20.39% (10,627/52,108), respectively. Additionally, disorders of lipoprotein metabolism (E78) showed high prevalence, at 15.29% (2800/18,311) in the private data set and 44.37% (23,120/52,108) in the MIMIC-IV data set.

Among comorbidities with significant differences (*P*<.05) in prevalence, the differences were substantial, with some comorbidities enriched in specific subgroups (Table 2). In the private data set, diabetes (E11), cerebrovascular diseases (I63 and I69), heart diseases (I11, I20, I25, I48, and I50), chronic obstructive pulmonary disease (J43 and J44), renal diseases (N04, N18, N20, and N28), and gout (M10) were enriched in males. Conversely, systemic connective tissue disorders (M35), dorsopathies (M48 and M50), and disorders of bone density and structure (M80 and M81) were enriched in females.

Most comorbidities had higher prevalence and were even enriched in patients with BTD, including heart disease (I20, I25, and I50), cerebrovascular disease (I63, I65, and I67), hypertension (I10), diabetes (E11), liver disease (K74), gastritis and duodenitis (K29), and renal disease (N18, N20, and N08). Notably, malignant neoplasms were enriched in the TC group, including nasopharyngeal carcinoma (C11), bronchus and lung cancer (C34), and lymph node cancers (C77). Tables S3 and S4 in Multimedia Appendix 1 present the complete prevalence differences of enriched comorbidities in the subgroups of patients with TD from the private and MIMIC-IV data sets, respectively.

**Figure 5:**
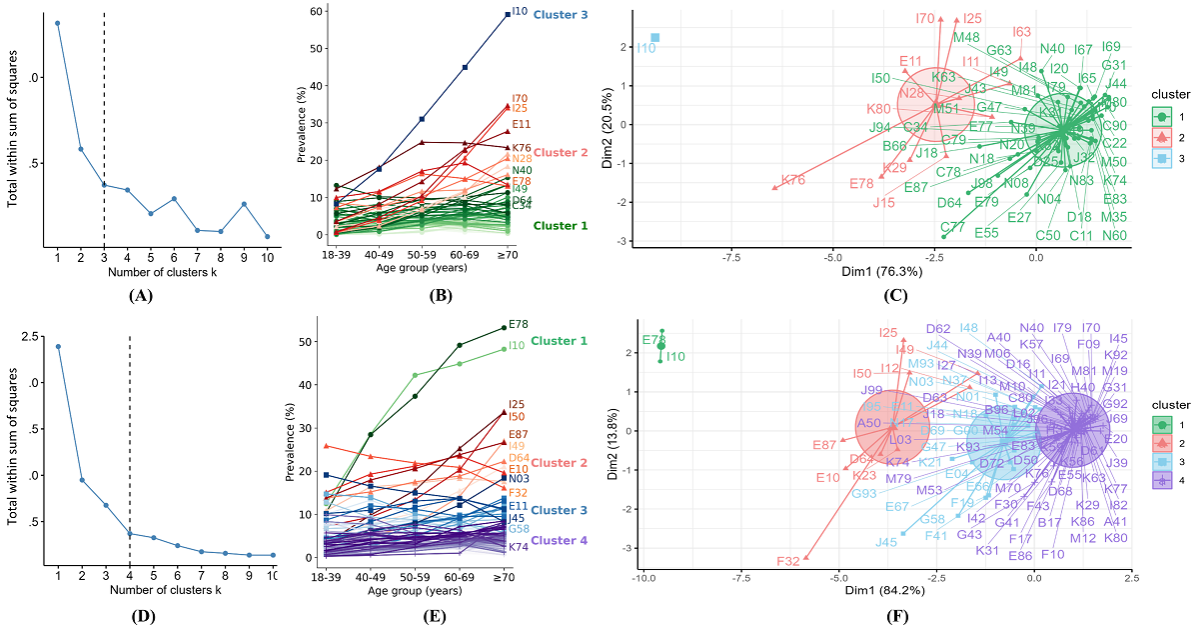
(A) and (D) The optimal number of clusters identified using the K-means clustering algorithm. (B) and (E) The age-specific prevalence of comorbidities within each cluster. (C) and (F) The cluster plots, where comorbidity prevalence in the 5 age groups (18-39, 40-49, 50-59, 60-69, and ≥70 years) has been reduced to 2 dimensions (x-axis and y-axis) through principal component analysis. Panels A-C are based on the private data set, while panels D-F correspond to the MIMIC-IV data set. A detailed list of ICD-10 codes is provided in Tables S1 and S2 in Multimedia Appendix 1. ICD-10: International Classification of Diseases, 10th Revision; MIMIC: Medical Information Mart for Intensive Care.

Age-specific comorbidities were clustered into 3 groups in the private data set and 4 groups in the MIMIC-IV data set (Figure 5). Most comorbidities’ prevalence generally increased with age, although the rates varied by cluster. In the private data set, cluster 1 comprised 55 comorbidities with low, mostly stable prevalence. Cluster 2 included 11 comorbidities with moderate prevalence and high growth rates, such as atherosclerosis (I70), ischemic heart disease (I25), and diabetes (E11). Cluster 3 contained a single comorbidity (hypertension, I10) with the highest growth rate with age. In the MIMIC-IV data set, cluster 1 included 2 comorbidities (lipoprotein metabolism disorder, E78, and hypertension, I10) that showed significant age-related increases. Cluster 2 had 9 comorbidities with moderate increases, while clusters 3 and 4 comprised 22 and 70 comorbidities with low prevalence, respectively.

**Table 2:** Absolute prevalence differences of enrichment comorbidities in subgroups of patients with thyroid diseases on the private data set.

ICD-10^a^ code	Enrichment comorbidity	Crude prevalence (95% CI)	Absolute prevalence difference, value (95% CI)	
			Male – Female	TC^b^ – BTD^c^
A49	Bacterial infection	2.7 (2.5 to 3.0)	N/A^d^	–2.6 (–3.0 to –2.2)
B66	Fluke infections	3.4 (3.1 to 3.6)	5.1 (4.5 to 5.7)	–3.0 (–3.5 to –2.5)
C11	Nasopharyngeal carcinoma	1.7 (1.5 to 1.9)	2.2 (1.8 to 2.6)	–1.7 (–2.0 to –1.4)
C22	Malignant neoplasm of the liver and intrahepatic bile ducts	1.7 (1.5 to 1.9)	1.8 (1.4 to 2.2)	–1.8 (–2.0 to –1.6)
C34	Bronchial and lung cancer	7.8 (7.4 to 8.1)	3.7 (2.9 to 4.5)	–7.5 (–8.1 to –6.9)
C50	Malignant neoplasm of the breast	2.4 (2.2 to 2.6)	–3.9 (–4.3 to –3.5)	–2.5 (–2.8 to –2.2)
C77	Secondary lymph node carcinoma	9.2 (8.8 to 9.7)	4.0 (3.1 to 4.9)	31.9 (29.0 to 34.8)
C78	Secondary malignant neoplasm of the respiratory and digestive organs	6.4 (6.1 to 6.8)	N/A	–3.0 (–4.1 to –1.9)
C79	Secondary malignant neoplasm	5.3 (5.0 to 5.6)	2.9 (2.2 to 3.6)	–3.2 (–4.1 to –2.3)
C90	Multiple myeloma and malignant plasma cell neoplasms	1.7 (1.5 to 1.9)	1.9 (1.5 to 2.3)	–1.8 (–2.0 to –1.6)
D18	Hemangioma and lymphangioma	3.1 (2.8 to 3.3)	N/A	–2.1 (–2.8 to –1.4)
D25	Leiomyoma of the uterus	3.3 (3.0 to 3.5)	–5.4 (–5.8 to –5.0)	–2.9 (–3.4 to –2.4)
D64	Anemias	8.5 (8.1 to 8.9)	N/A	–6.9 (–7.8 to –6.0)
E11	Diabetes mellitus	16.8 (16.3 to 17.4)	7.2 (6.1 to 8.3)	–14.6 (–15.8 to –13.4)
E27	Disorders of the adrenal gland	3.3 (3.1 to 3.6)	2.2 (1.6 to 2.8)	–3.4 (–3.8 to –3.0)
E55	Vitamin D deficiency	4.8 (4.5 to 5.2)	N/A	–5.0 (–5.4 to –4.6)
E77	Disorders of glycoprotein metabolism	4.4 (4.1 to 4.7)	N/A	–3.3 (–4.0 to –2.6)
E78	Disorders of lipoprotein metabolism	15.3 (14.8 to 15.8)	N/A	–12.5 (–13.7 to –11.3)
E79	Disorders of purine and pyrimidine metabolism	5.4 (5.1 to 5.7)	3.8 (3.1 to 4.5)	–3.1 (–4.1 to –2.1)
E83	Disorders of mineral metabolism	1.6 (1.4 to 1.8)	N/A	2.5 (1.3 to 3.7)
E87	Disorders of fluid balance	6.9 (6.6 to 7.3)	N/A	–3.5 (–4.7 to –2.3)
G31	Degenerative diseases of the nervous system	2.3 (2.0 to 2.5)	1.1 (0.6 to 1.6)	–2.4 (–2.6 to –2.2)
G47	Sleep disorders	2.6 (2.4 to 2.9)	N/A	–2.7 (–3.0 to –2.4)
G63	Polyneuropathy diseases	3.3 (3.0 to 3.5)	N/A	–3.4 (–3.7 to –3.1)
I10	Hypertension	35.1 (34.5 to 35.8)	N/A	–25.2 (–27.2 to –23.2)
I11	Hypertensive heart disease	9.3 (8.9 to 9.7)	4.2 (3.3 to 5.1)	–9.3 (–9.9 to –8.7)
I20	Angina pectoris	4.2 (3.9 to 4.5)	3.5 (2.9 to 4.1)	–4.4 (–4.8 to –4.0)
I25	Ischemic heart disease	14.9 (14.4 to 15.5)	8.1 (7.0 to 9.2)	–14.6 (–15.5 to –13.7)
I48	Atrial fibrillation and flutter	3.1 (2.9 to 3.4)	1.5 (1.0 to 2.0)	–3.2 (–3.5 to –2.9)
I49	Cardiac arrhythmias	5.3 (5.0 to 5.6)	N/A	–5.3 (–5.8 to –4.8)
I50	Heart failure	7.4 (7.0 to 7.7)	4.6 (3.8 to 5.4)	–7.8 (–8.2 to –7.4)
I63	Cerebral infarction	9.3 (8.9 to 9.7)	4.2 (3.3 to 5.1)	–9.8 (–10.3 to –9.3)
I65	Anterior cerebral artery stenosis and occlusion	3.0 (2.7 to 3.2)	N/A	–3.2 (–3.5 to –2.9)
I67	Cerebrovascular diseases	4.2 (3.9 to 4.5)	N/A	–4.3 (–4.7 to –3.9)
I69	Sequelae of cerebrovascular disease	2.9 (2.7 to 3.1)	2.3 (1.8 to 2.8)	–2.8 (–3.2 to –2.4)
I70	Atherosclerosis	16.1 (15.5 to 16.6)	N/A	–15.3 (–16.2 to –14.4)
I79	Disorders of arteries, arterioles, and capillaries	3.6 (3.3 to 3.9)	N/A	–3.9 (–4.2 to –3.6)
J15	Bacterial pneumonia	11.6 (11.1 to 12.0)	N/A	–11.8 (–12.4 to –11.2)
J18	Pneumonia	9.2 (8.8 to 9.7)	N/A	–5.8 (–7.0 to –4.6)
J32	Chronic sinusitis	3.0 (2.8 to 3.3)	1.6 (1.1 to 2.1)	–2.4 (–3.0 to –1.8)
J43	Emphysema	4.1 (3.8 to 4.3)	5.1 (4.4 to 5.8)	–2.7 (–3.5 to –1.9)
J44	Chronic obstructive pulmonary disease	1.9 (1.7 to 2.1)	3.2 (2.7 to 3.7)	–2.1 (–2.3 to –1.9)
J94	Pleural conditions	4.1 (3.8 to 4.4)	N/A	–3.9 (–4.4 to –3.4)
K29	Gastritis and duodenitis	13.5 (13.0 to 14.0)	N/A	–13.4 (–14.2 to –12.6)
K31	Diseases of the stomach and duodenum	2.9 (2.7 to 3.1)	N/A	–3.0 (–3.3 to –2.7)
K63	Diseases of intestine	2.6 (2.3 to 2.8)	N/A	–2.7 (–2.9 to –2.5)
K74	Fibrosis and cirrhosis of the liver	2.3 (2.1 to 2.5)	1.7 (1.2 to 2.2)	–1.8 (–2.3 to –1.3)
K76	Liver disease	22.0 (21.4 to 22.6)	N/A	–17.4 (–18.9 to –15.9)
K80	Cholelithiasis	9.2 (8.7 to 9.6)	N/A	–7.7 (–8.6 to –6.8)
K82	Diseases of the gallbladder	3.3 (3.1 to 3.6)	N/A	–1.7 (–2.5 to –0.9)
M10	Gout	2.4 (2.2 to 2.6)	4.6 (4.1 to 5.1)	–2.2 (–2.6 to –1.8)
M35	Systemic involvement of connective tissue	2.0 (1.8 to 2.2)	–2.5 (–2.8 to –2.2)	–2.0 (–2.3 to –1.7)
M48	Spondylopathies	4.8 (4.5 to 5.1)	–2.1 (–2.7 to –1.5)	–4.8 (–5.3 to –4.3)
M50	Cervical disc disorders	2.4 (2.2 to 2.6)	–1.3 (–1.7 to –0.9)	–2.4 (–2.7 to –2.1)
M51	Intervertebral disc disorders	4.0 (3.7 to 4.3)	N/A	–4.0 (–4.4 to –3.6)
M80	Osteoporosis with pathological fracture	1.9 (1.7 to 2.1)	–1.6 (–2.0 to –1.2)	–1.9 (–2.2 to –1.6)
M81	Osteoporosis without pathological fracture	7.1 (6.7 to 7.4)	–4.2 (–4.9 to –3.5)	–7.2 (–7.7 to –6.7)
N04	Nephrotic syndrome	3.0 (2.8 to 3.3)	3.1 (2.5 to 3.7)	–3.2 (–3.5 to –2.9)
N08	Glomerular disorders	4.1 (3.8 to 4.3)	N/A	–4.2 (–4.6 to –3.8)
N18	Chronic renal failure	5.5 (5.1 to 5.8)	4.5 (3.8 to 5.2)	–5.6 (–6.0 to –5.2)
N20	Calculus of the kidney and ureter	5.4 (5.0 to 5.7)	3.0 (2.3 to 3.7)	–4.2 (–5.0 to –3.4)
N28	Disorders of the kidney and ureter	12.1 (11.6 to 12.6)	6.0 (5.0 to 7.0)	–11.2 (–12.1 to –10.3)
N39	Disorders of the urinary system	3.0 (2.8 to 3.3)	–1.5 (–2.0 to –1.0)	–3.2 (–3.5 to –2.9)
N40	Hyperplasia of the prostate	7.5 (7.1 to 7.9)	19.2 (18.3 to 20.1)	–7.7 (–8.2 to –7.2)
N60	Benign mammary dysplasia	1.5 (1.3 to 1.7)	–2.5 (–2.8 to –2.2)	N/A

^a^ICD-10: International Classification of Diseases, 10th Revision.

^b^TC: thyroid cancer.

^c^BTD: benign thyroid disease.

^d^Not applicable, as the comorbidity was not enriched in this subgroup.

### Phenotypic Comorbidity Network in Patients With Thyroid Disease

**Figure 6:**
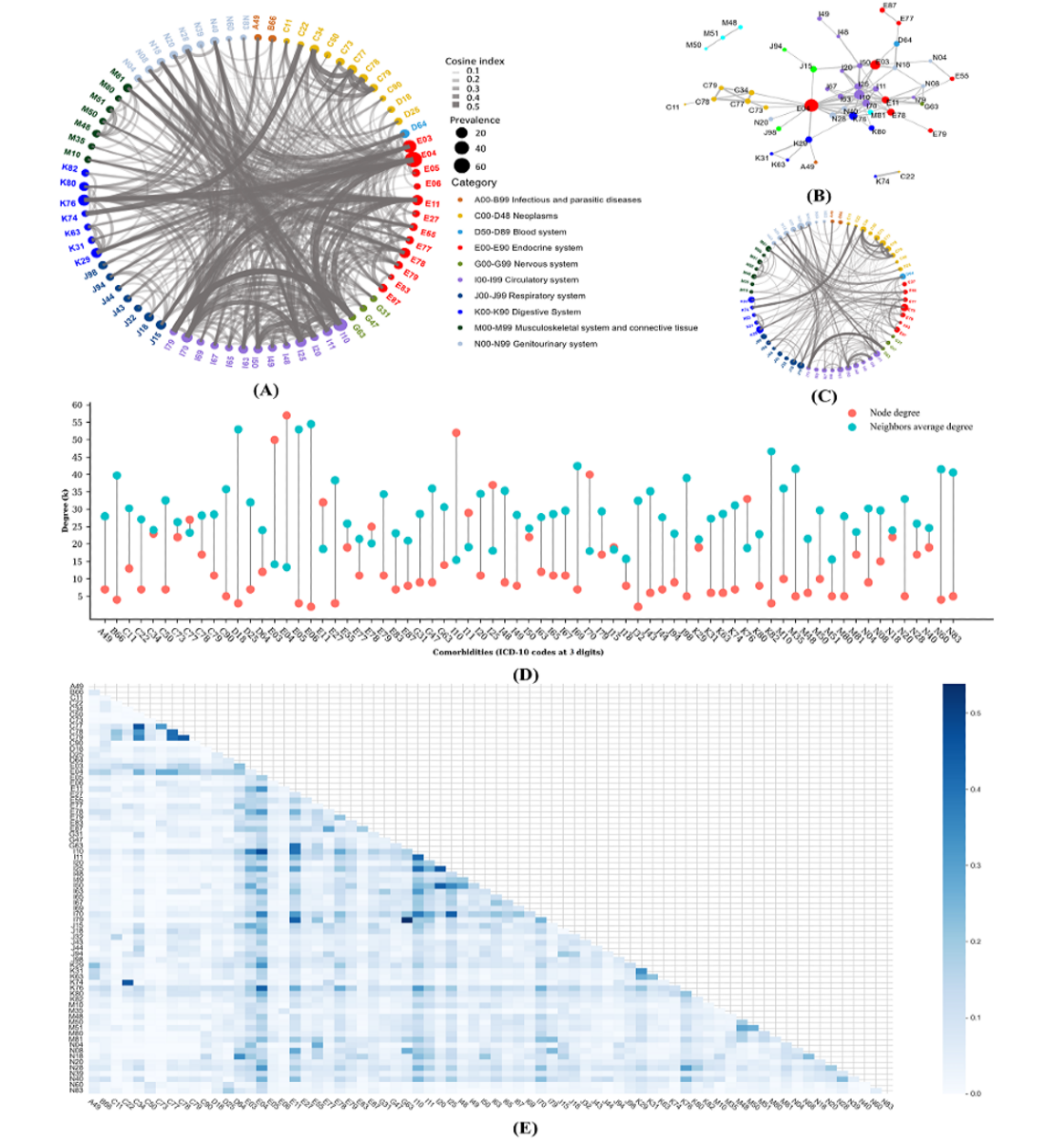
Phenotypic comorbidity network (PCN) in patients with thyroid disease (TD) on the private data set. (A) The PCN of patients with TD. Nodes represent comorbidities. The node size is proportional to the comorbidity prevalence in patients with TD and its color is used to identify the disease category. Link weights are proportional to the magnitudes of the cosine index which are >0. (B) The 103 edges in the PCN, for which the cosine index values are ≥0.20. (C) PCN of dropping the top 7 diseases with the highest degree and the highest betweenness centrality. (D) The degree distribution of the node and its neighbors. (E) Correlations measured by the cosine index between diseases. The disease codes are listed in Table S1 in Multimedia Appendix 1.

In the private data set, the PCN of patients with TD contained 72 diseases and 492 disease pairs (Figure 6A). Each disease shared a median of 13.7 significant correlations with other comorbidities. Nontoxic goiter (E04), hypothyroidism (E03), and TC (C73) were prevalent TDs, with degrees of 50, 57, and 22, respectively (Figure 6D). Hypothyroidism (E03) was strongly linked to hypertension (I10), bacterial pneumonia (J15), heart failure (I50), ischemic heart disease (I25), and anemias (D64), with cosine indexes ranging from 0.23 to 0.36 (Figure 6A and 6E). By contrast, nontoxic goiter (E04) was associated with hypertension (I10; cosine index=0.47), liver disease (K76; cosine index=0.44), atherosclerosis (I70; cosine index=0.37), gastritis and duodenitis (K29; cosine index=0.33), and disorders of the renal and ureter (N28; cosine index=0.33), differing from those of hypothyroidism (E03). Nontoxic goiter (E04) and secondary lymph node carcinoma (C77) showed the highest correlation with TC (C73), with cosine indexes of 0.32 and 0.27, respectively, while other comorbidities had a cosine index of less than 0.06 with TC.

In the private data set, hypertension (I10), atherosclerosis (I70), ischemic heart disease (I25), diabetes (E11), and liver diseases (K76) were the top 5 comorbidities with high degrees (range 32-52) and betweenness centrality (range 47.1-385.2), reflecting their frequent coexistence and mediating role in disease correlations. Among the top 103 comorbid disease pairs with a cosine index of 0.20 or higher, some connections were within the same disease system, such as 50 and 12 connections within circulatory system diseases (ICD-10 codes starting with I) and malignant neoplasms (ICD-10 codes starting with C). When the diseases mentioned above (ie, E03, E04, I10, I70, I25, E11, and K76), which account for about 10% (7/72) of the diseases in the PCN, were removed, the scale of the PCN (Figure 6C) decreased significantly, with the number of significant comorbid disease pairs dropping by 57.5% (283/492).

In the MIMIC-IV data set, there were 107 diseases with 1819 disease links in the PCN, as shown in Figure S2 in Multimedia Appendix 1. Hypertension (I10), ischemic heart disease (I25), heart failure (I50), diabetes (E10 and E11), and esophageal disease (K23) were closely connected with TDs, with the highest cosine index values of 0.60, 0.48, 0.46, 0.42, and 0.39, respectively. Figures S3 and S4 in Multimedia Appendix 1 display the PCN for each subgroup in the private and MIMIC-IV data sets, respectively.

### Differences in Phenotypic Comorbidity Networks Between Subgroups

In both data sets, the comorbidity coexistence relationships were more complex in males than in females and in patients with BTD compared with those with TC (Table 3). In the private data set, males had 60 comorbidities and females had 63, with the median numbers of connections for comorbidities of 14.0 and 11.5, respectively. Patients with TC had fewer comorbidities (lower degrees) than those with BTD. As patients aged, the PCNs exhibited an increasing average degree of nodes and their neighboring nodes across the 5 age groups, indicating increasingly complex comorbidity coexistence relationships.

**Figure 7:**
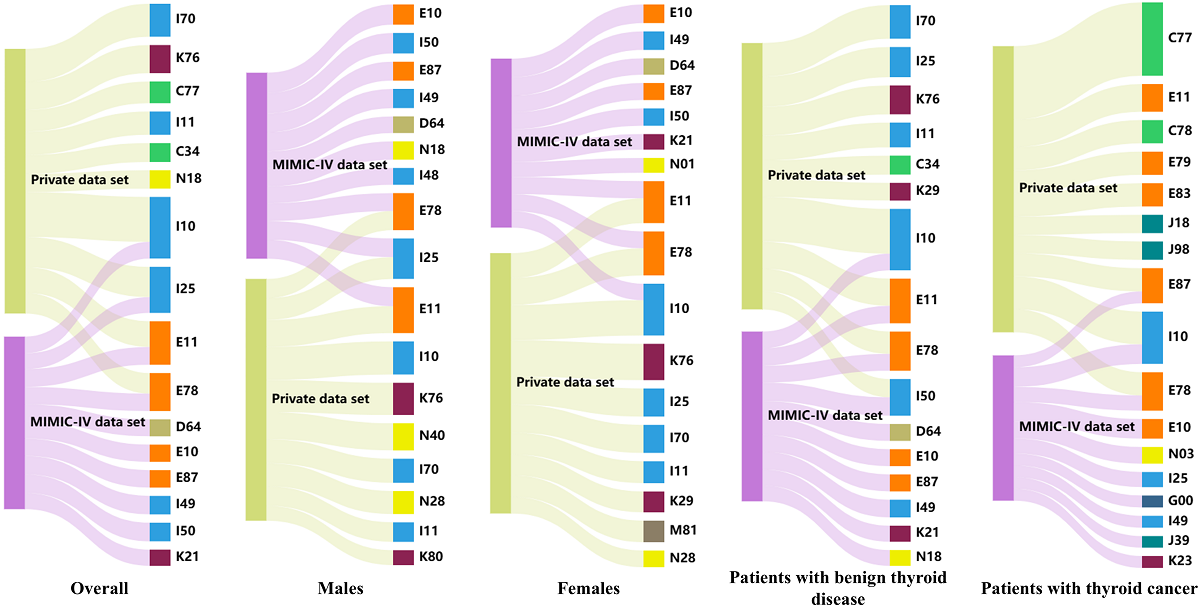
Sankey diagrams of the top 10 important comorbidities of patients with thyroid disease in subgroups on the 2 data sets. The links between the subgroups and comorbidities were proportional to the PageRank values. The higher the PageRank value, the stronger the connection, and the more significant the comorbidity. MIMIC: Medical Information Mart for Intensive Care.

Figure 7 displays the top 10 important comorbidities with the highest PageRank values in different patient groups. In both data sets, cardiovascular and cerebrovascular diseases (colored in blue, ICD-10 codes with I), such as hypertension (I10 and I11) and ischemic heart disease (I25), along with endocrine and metabolic diseases (colored in orange, ICD-10 codes with E), such as diabetes (E10 and E11) and lipoprotein metabolism disorder (E78), showed high PageRank values, indicating their significance in the PCN of patients with TD. Notably, secondary lymph node carcinoma (C77) exhibited a particularly high PageRank value within the PCN of patients with TD in the private data set, especially among patients with TC.

**Figure 8:**
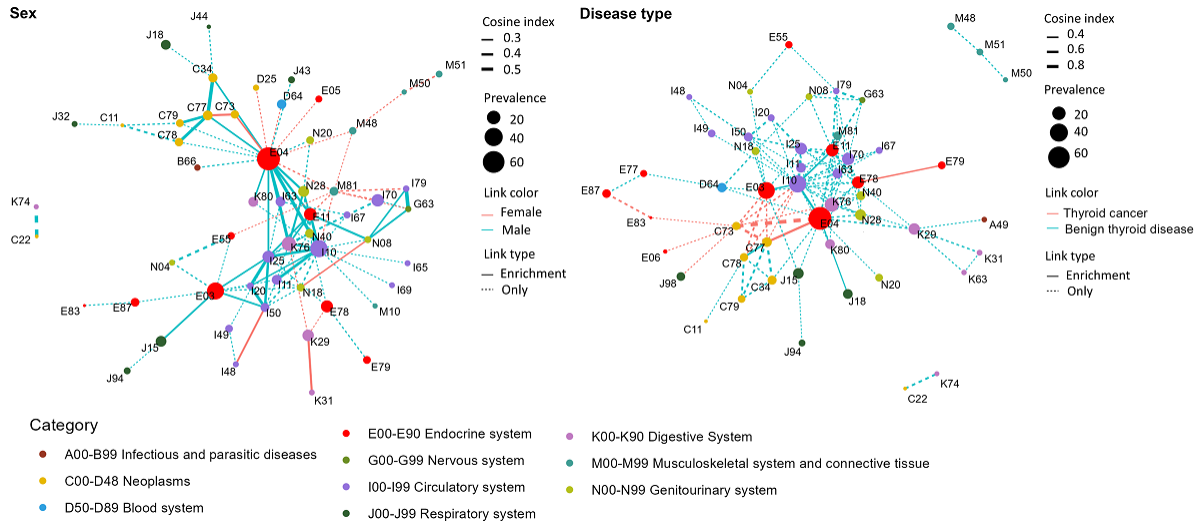
Abundant connections by sex and disease type in patients with thyroid disease on the private data set. Except for those common to both subgroups (link weight difference, ie, cosine index <0.05), disease pairs were identified as abundant edges in 1 subgroup, including enrichment (solid lines) or only occurrence (dotted lines).

Figure 8 illustrates the abundant connections among patients with TD in the private data set. In the sex-specific PCN, there were 103 connections involving 55 comorbidities common to both sexes. Abundant connections in females were primarily linked to atrial fibrillation and flutter (I48), heart failure (I50), diseases of the stomach and duodenum (K29 and K31), and renal failure (N18). By contrast, abundant connections in males were mainly associated with various cardiocerebrovascular diseases (ICD-10 codes with I), malignant neoplasms (ICD-10 codes with C), bacterial pneumonia (J15), disorders of the ureter (N28), and cholelithiasis (K80). Differences in connections based on disease type were relatively minor, with 2 abundant connections in patients with TC and 5 in those without. The abundant connections in patients with TC were primarily linked to metabolic disorders (E78 and E79). By contrast, patients with BTD exhibited several abundant connections, including hypertension (I10), diabetes (E11), pneumonia (J18), and cholelithiasis (K80). The abundant connections within a specific subgroup of patients with TD from the MIMIC-IV data set are shown in Figure S5 in Multimedia Appendix 1.

**Table 3:** Phenotypic comorbidity network structures in subgroups of patients with thyroid disease.

Subgroup		Nodes, n		Density		Degree, median (IQR)			Degree of neighbors, median (IQR)		
		MIMIC^a^-IV	Private	MIMIC-IV	Private	MIMIC-IV	Private	MIMIC-IV		Private	
All		102	72	0.321	0.192	30.0 (20.0-46.0)	9.0 (6.0-17.5)	49.2 (44.0-54.9)		28.3 (23.1-34.4)	
**Sex**											
	Female	90	60	0.312	0.240	27.0 (18.0-40.0)	11.5 (6.0-16.0)	43.0 (36.7-46.3)		26.7 (21.3-30.7)	
	Male	92	63	0.229	0.304	17.0 (10.8-27.8)	14.0 (10.0-22.0)	38.7 (33.2-43.0)		32.9 (28.8-39.4)	
**Disease subgroup**											
	TC^b^	28	15	0.087	0.419	1.5 (1.0-2.0)	4.0 (3.5-5.5)	24.2 (16.5-31.0)		11.0 (8.8-12.2)	
	BTD^c^	100	69	0.338	0.198	30.0 (21.5-46.0)	10.0 (6.0-16.0)	48.4 (42.9-52.7)		28.3 (23.6-34.6)	
	Nontoxic goiter	81	65	0.111	0.217	6.0 (2.2-9.8)	10.0 (6.0-15.0)	31.2 (24.7-46.7)		30.9 (22.2-34.0)	
	Hypothyroidism	100	61	0.329	0.237	29.0 (20.0-43.0)	11.0 (7.0-17.0)	47.0 (41.4-51.8)		28.2 (23.3-33.7)	
	Hyperthyroidism	79	39	0.098	0.208	4.0 (2.0-9.2)	5.0 (3.0-8.0)	30.8 (21.6-46.1)		23.0 (18.6-29.8)	
**Age group (years)**											
	18-39	57	46	0.115	0.133	6.0 (2.0-9.0)	4.0 (3.0-6.0)	11.9 (10.0-14.3)		21.1 (15.8-29.7)	
	40-49	71	55	0.127	0.143	7.0 (4.0-15.2)	5.0 (3.0-7.0)	17.5 (14.6-20.1)		27.0 (22.6-33.0)	
	50-59	85	66	0.171	0.207	12.0 (6.0-21.0)	9.5 (6.0-14.0)	25.7 (23.0-30.1)		33.7 (26.7-39.1)	
	60-69	92	65	0.206	0.265	17.0 (9.0-25.5)	12.0 (8.0-19.0)	34.6 (29.2-39.5)		35.7 (28.9-42.6)	
	≥70	89	59	0.265	0.353	22.0 (10.0-35.0)	15.0 (11.0-22.5)	39.2 (33.9-43.5)		38.2 (32.0-41.2)	

^a^MIMIC: Medical Information Mart for Intensive Care.

^b^TC: thyroid cancer.

^c^BTD: benign thyroid disease.

## Discussion

### Principal Findings

In this population-based cohort study, we used a network-based approach to reveal the comprehensive comorbidity patterns in patients with TD, highlighting differences across sex, age, and TD types. Comorbidities were prevalent among patients with TD, with variations in prevalence and complex coexistence relationships influenced by sex, age, and the type of TD. Nontoxic goiter, hypothyroidism, hyperthyroidism, and TC were identified as the most common TDs. The top 5 comorbidities with the highest prevalence were hypertension, liver disease, diabetes, atherosclerosis, and disorders of lipoprotein metabolism. The number of comorbidities increased with age across all patients with TD and subgroups. Males and patients with BTD demonstrated higher rates of comorbidity, greater disease diversity, and stronger associations compared with females and patients with TC.

In this study, hypertension emerged as the most significant comorbidity, affecting 35.14% (6435/18,311) of patients with TD in the private data set. A previous study has demonstrated a positive correlation between blood pressure and thyroid-stimulating hormone levels [17]. Additionally, both blood pressure and dyslipidemia have been strongly associated with TNs [18]. Consistent with previous studies [5,19-22], we found a strong link between TD and diabetes. Specific thyroid disorders, such as TNs [22], hypothyroidism [19], thyroid dysfunction [5], and thyroid disorders [20], have been linked to diabetes. Furthermore, thyroid function significantly impacts the cardiovascular system [23]. Both hyperthyroidism and hypothyroidism are associated with increased cardiovascular morbidity and mortality, and abnormal thyroid function elevates the risk of coronary heart disease [6,24]. Our findings also revealed high PageRank values for ischemic heart diseases within the PCN of patients with TD. Additionally, thyroid hormones regulate metabolism, linking metabolic syndrome and thyroid dysfunction to significant morbidity and mortality [23]. We also observed that metabolic disorders, such as disorders of lipoprotein metabolism, were closely associated with patients with TD. Moratalla et al [8], exploring the comorbidity network of hypothyroidism, showed strong links to thyroid/larynx cancer, hyperthyroidism, anemia, and goiter. We similarly found associations with nasopharyngeal carcinoma, secondary respiratory cancers, anemia, hyperthyroidism, and nontoxic goiter. However, we revealed that hypothyroidism was strongly associated with hypertension and pneumonia, which was unreported in the study by Moratalla et al [8].

In this study, females exhibited a higher TD burden than males, especially among younger individuals. Similar findings have been widely reported in several studies [22,25]. Consistent with previous studies [22,25], TDs were more prevalent in females than males [1], and the incidence of TC was significantly higher among females, with a ratio of 2.36 in 2017 [26]. This could be attributed to factors such as physiology, pregnancy, and estrogen exposure [27,28]. Estrogen, which is higher in females, can lead to thyroid gland changes, increasing the risk of TDs [27]. Therefore, greater attention should be paid to thyroid gland examination in females.

Further, our findings revealed sex-specific comorbidity patterns in patients with TD. Males, despite lower TD incidence, tended to have more comorbidities than females, with 31 enriched disease pairs in males’ PCN compared with only 5 in females on the private data set. Moratalla et al [8] also found that comorbidity networks showed more and stronger connections in males than in females. Notably, cancers, especially malignant neoplasms of lymph nodes, bronchus and lung, and respiratory and digestive organs, demonstrated enriched connections with TC in the PCN of males. Moreover, despite evidence of higher primary TC incidence among females [26,29], males faced a greater risk of developing second primary TC compared with females [30-32]. For instance, males had a higher risk of head and neck cancer than females [30]. Our results confirmed that males were more likely to have concurrent cancers other than TC than females. Besides, males exhibited enrichment in multiple cardiocerebrovascular diseases, while females displayed fewer heart disease associations. Understanding these sex-specific comorbidity patterns aids in developing tailored treatment plans for patients with TD.

Our findings showed that older patients with TD, regardless of sex or TD type, tend to have more comorbidities, which increase with age. This aligns with previous research, including Stenholm et al [33], who reported a rise in diseases and physical difficulties with age, and Xu et al [22], who found a similar trend in TNs’ prevalence. Therefore, enhanced nursing and care for older adult patients with TD is crucial to reduce comorbidities, improve quality of life, and ease medical burdens.

Notably, the average age of patients with TC in this study was 44 years, the same for both genders. A previous study [34] also found that 45-year-old women are the most prone to papillary thyroid carcinoma, while another study [26] reported peak ages of 50-69 years for males and 15-49 years for females. However, Huang et al [2] found that the incidence of TC increased in populations aged under 40 years in China, with males showing a higher growth rate (18.6 vs 13.3). Some researchers suggested that young patients may delay seeking treatment due to underestimating the cancer risk [35]. We speculated that high workload and mental stress in young people may cause endocrine issues and hormone imbalances, contributing to TC’s younger trend. Given the link between neuropsychological functions and thyroid health [36,37], extra care is needed for young patients with TD to prevent cancer.

Besides, we observed that patients with TC, despite having a relatively low median comorbidity count (3 and 4 on the private and MIMIC-IV data sets, respectively), exhibited severe comorbidities such as cancer, heart failure, cerebral infarction, and pneumonia. This severity may stem from the aggressive nature of cancer and its tendency to metastasize, leading to life-threatening complications. Prior studies have shown higher risks of papillary TC following other neoplasms, particularly renal and breast cancers, as well as leukemias/lymphomas [38,39]. Similarly, Lian et al [40] found that subclinical hypothyroidism was common in patients with nasopharyngeal carcinoma within a year and that thyroid volume is a risk factor for radiation-induced hypothyroidism. Given these findings, heightened vigilance is necessary to prevent cancer progression and deterioration in patients with TC.

From a methodological perspective, we used cosine similarity and Pearson correlation indices to quantify node connectivity, considering disease prevalence and minimizing sample size biases. Cosine similarity effectively captured significant comorbidities in this study, as well as in previous analyses of comorbidities associated with colorectal cancer [10] and idiopathic cardiomyopathy [41]. Other similarity metrics, such as the Jaccard index [42], which quantifies the overlap between disease sets, and the observed-to-expected ratio, which considers the observed prevalence of disease pairs relative to the expected prevalence [7], were utilized in constructing cardiovascular comorbidity networks [7,42]. Additionally, various network properties were used to computationally identify key comorbidities. We applied the PageRank algorithm, which considers the number and weight of edges, the number of neighbor edges, and the centrality of neighbors, to assess the importance of comorbidities. In the comorbidity networks of both data sets in this study, the comorbidities selected by PageRank were similar, including hypertension and diabetes. PageRank has also been utilized in constructing comorbidity networks for patients with colorectal cancer [10], general patients [16], and hypothyroidism patients [8]. Additionally, other properties, such as degree [11] and degree centrality [42], have been used to identify important comorbidities and their connections.

### Limitations

This study has several limitations. First, apart from the MIMIC-IV data set, we collected data and conducted analyses solely in a general tertiary hospital in China, which may introduce selection bias, particularly concerning geographical and population factors. Utilizing multicenter data could yield more representative and robust results. Second, our analysis focused solely on the coexistence of comorbidities without considering their causal relationships, and many network properties remain unexplained in clinical or biological contexts. Some diseases may share common pathophysiological mechanisms or causes, leading to mutual influence. In some cases, one disease may promote the occurrence and progression of another. Therefore, further analysis is needed to explore the deeper and more detailed causal relationships between thyroid disorders and their comorbidities. Third, relying solely on ICD-10 codes for disease identification may not fully capture all cases related to thyroid disorders, potentially leading to missing information. Additionally, some diagnoses may be subject to overdiagnosis or underdiagnosis due to the complexity of clinical issues. Certain diseases may not be frequently coded, and broad categorization may obscure differences among heterogeneous conditions. Finally, we conducted only a cross-sectional analysis and did not consider the temporality and sequence of comorbidities when constructing comorbidity profiles. These patterns may change as diseases progress. By collecting more data over longer periods from multiple hospitals, a more detailed analysis could provide deeper insights into the comorbidity patterns of patients with thyroid disorders.

### Conclusions

This data-driven exploration of comorbidity patterns among all patients with thyroid disorder and their subgroups aims to provide valuable clinical and foundational insights into the comorbidities associated with thyroid disorders. It enhances our understanding of thyroid disorders as a whole and may inform the development of more effective and integrated therapeutic strategies for patients. Additionally, the network-based methodology utilized in this study has the potential to uncover comorbidity patterns in other diseases. In the future, we plan to incorporate a wider variety of data to construct more comprehensive networks. By extracting features from these networks, we aim to leverage medical knowledge to develop machine learning–based prediction models for patients with thyroid disorders.

## Appendix

Multimedia Appendix 1 Additional data analysis.

## Abbreviations

BTD: benign thyroid disease
EMR: electronic medical record
ICD-10: International Classification of Diseases, 10th Revision
MIMIC: Medical Information Mart for Intensive Care
PCN: phenotypic comorbidity network
TC: thyroid cancer
TD: thyroid disease
TN: thyroid nodule

## Data Availability

The MIMIC-IV data sets used in this study are available online [43]. However, the private data set generated or analyzed in this study is not publicly available as these are restricted by the Medical Ethics Committee of the First Affiliated Hospital of Guangxi Medical University.
