# Supplementary material for: Analyzing Comorbidity Patterns in Patients With Thyroid Disease Using Large-Scale Electronic Medical Records: Network-Based Retrospective Observational Study
Source: Interact J Med Res. 2024 Oct 3;13:e54891. doi: 10.2196/54891 (PMC11487213; doi:10.2196/54891)
Supplement: Multimedia Appendix 1 [file ijmr_v13i1e54891_app1.docx]

Table S1. The frequency and prevalence of diseases in patients with thyroid disease on the private dataset.

| ICD-10^a^ | Disease name | Number of disease | Prevalence,% (95%CI^b^) |
| --- | --- | --- | --- |
| A49 | Bacterial infection | 501 | 2.7(2.5, 3.0) |
| B66 | Fluke infections | 620 | 3.4(3.1, 3.6) |
| C11 | Malignant neoplasm of nasopharynx | 309 | 1.7(1.5, 1.9) |
| C22 | Malignant neoplasm of liver and intrahepatic bile ducts | 314 | 1.7(1.5, 1.9) |
| C34 | Malignant neoplasm of bronchus and lung | 1420 | 7.8(7.4, 8.1) |
| C50 | Malignant neoplasm of breast | 440 | 2.4(2.2, 2.6) |
| C73 | Malignant neoplasm of thyroid gland | 1144 | 6.2(5.9, 6.6) |
| C77 | Secondary malignant neoplasm of lymph nodes | 1691 | 9.2(8.8, 9.7) |
| C78 | Secondary malignant neoplasm of respiratory and digestive organs | 1180 | 6.4(6.1, 6.8) |
| C79 | Secondary malignant neoplasm | 966 | 5.3(5.0, 5.6) |
| C90 | Multiple myeloma and malignant plasma cell neoplasms | 313 | 1.7(1.5, 1.9) |
| D18 | Haemangioma and lymphangioma | 562 | 3.1(2.8, 3.3) |
| D25 | Leiomyoma of uterus | 602 | 3.3(3.0, 3.5) |
| D64 | Anaemias | 1563 | 8.5(8.1, 8.9) |
| E03 | Hypothyroidism | 6217 | 34.0(33.3, 34.6) |
| E04 | Nontoxic goitre | 12410 | 67.8(67.1, 68.5) |
| E05 | Hyperthyroidism | 761 | 4.2(3.9, 4.4) |
| E06 | Thyroiditis | 351 | 1.9(1.7, 2.1) |
| E11 | Diabetes mellitus | 3080 | 16.8(16.3, 17.4) |
| E27 | Disorders of adrenal gland | 612 | 3.3(3.1, 3.6) |
| E55 | Vitamin D deficiency | 887 | 4.8(4.5, 5.2) |
| E77 | Disorders of glycoprotein metabolism | 803 | 4.4(4.1, 4.7) |
| E78 | Disorders of lipoprotein metabolism | 2800 | 15.3(14.8, 15.8) |
| E79 | Disorders of purine and pyrimidine metabolism | 990 | 5.4(5.1, 5.7) |
| E83 | Disorders of mineral metabolism | 297 | 1.6(1.4, 1.8) |
| E87 | Disorders of fluid, electrolyte and acid-base balance | 1271 | 6.9(6.6, 7.3) |
| G31 | Degenerative diseases of nervous system | 414 | 2.3(2.0, 2.5) |
| G47 | Sleep disorders | 484 | 2.6(2.4, 2.9) |
| G63 | Polyneuropathy diseases | 602 | 3.3(3.0, 3.5) |
| I10 | Hypertension | 6435 | 35.1(34.5, 35.8) |
| I11 | Hypertensive heart disease | 1702 | 9.3(8.9, 9.7) |
| I20 | Angina pectoris | 768 | 4.2(3.9, 4.5) |
| I25 | Ischemic heart disease | 2737 | 14.9(14.4, 15.5) |
| I48 | Atrial fibrillation and flutter | 569 | 3.1(2.9, 3.4) |
| I49 | Cardiac arrhythmias | 972 | 5.3(5.0, 5.6) |
| I50 | Heart failure | 1349 | 7.4(7.0, 7.7) |
| I63 | Cerebral infarction | 1703 | 9.3(8.9, 9.7) |
| I65 | Occlusion and stenosis of precerebral arteries | 541 | 3.0(2.7, 3.2) |
| I67 | Cerebrovascular diseases | 772 | 4.2(3.9, 4.5) |
| I69 | Sequelae of cerebrovascular disease | 530 | 2.9(2.7, 3.1) |
| I70 | Atherosclerosis | 2944 | 16.1(15.5, 16.6) |
| I79 | Disorders of arteries, arterioles and capillaries | 662 | 3.6(3.3, 3.9) |
| J15 | Bacterial pneumonia | 2116 | 11.6(11.1, 12.0) |
| J18 | Pneumonia | 1692 | 9.2(8.8, 9.7) |
| J32 | Chronic sinusitis | 550 | 3.0(2.8, 3.3) |
| J43 | Emphysema | 744 | 4.1(3.8, 4.3) |
| J44 | Chronic obstructive pulmonary disease | 352 | 1.9(1.7, 2.1) |
| J94 | Pleural conditions | 757 | 4.1(3.8, 4.4) |
| J98 | Respiratory disorders | 1286 | 7.0(6.7, 7.4) |
| K29 | Gastritis and duodenitis | 2478 | 13.5(13.0, 14.0) |
| K31 | Diseases of stomach and duodenum | 532 | 2.9(2.7, 3.1) |
| K63 | Diseases of intestine | 467 | 2.6(2.3, 2.8) |
| K74 | Fibrosis and cirrhosis of liver | 417 | 2.3(2.1, 2.5) |
| K76 | Liver disease | 4023 | 22.0(21.4, 22.6) |
| K80 | Cholelithiasis | 1676 | 9.2(8.7, 9.6) |
| K82 | Diseases of gallbladder | 606 | 3.3(3.1, 3.6) |
| M10 | Gout | 440 | 2.4(2.2, 2.6) |
| M35 | Systemic involvement of connective tissue | 359 | 2.0(1.8, 2.2) |
| M48 | Spondylopathies | 879 | 4.8(4.5, 5.1) |
| M50 | Cervical disc disorders | 437 | 2.4(2.2, 2.6) |
| M51 | Intervertebral disc disorders | 736 | 4.0(3.7, 4.3) |
| M80 | Osteoporosis with pathological fracture | 344 | 1.9(1.7, 2.1) |
| M81 | Osteoporosis without pathological fracture | 1294 | 7.1(6.7, 7.4) |
| N04 | Nephrotic syndrome | 552 | 3.0(2.8, 3.3) |
| N08 | Glomerular disorders | 744 | 4.1(3.8, 4.3) |
| N18 | Chronic renal failure | 999 | 5.5(5.1, 5.8) |
| N20 | Calculus of kidney and ureter | 983 | 5.4(5.0, 5.7) |
| N28 | Disorders of kidney and ureter | 2216 | 12.1(11.6, 12.6) |
| N39 | Disorders of urinary system | 555 | 3.0(2.8, 3.3) |
| N40 | Hyperplasia of prostate | 1368 | 7.5(7.1, 7.9) |
| N60 | Benign mammary dysplasia | 279 | 1.5(1.3, 1.7) |
| N83 | Noninflammatory disorders of ovary, fallopian tube and broad ligament | 284 | 1.6(1.4, 1.7) |

^a^ICD-10: International Classification of Diseases codes, 10th Revision

^b^CI: confidence interval.

Table S2. The frequency and prevalence of diseases in patients with thyroid disease on the MIMIC-IV dataset.

| ICD-10^a^ | Disease name | | Number of disease | | Prevalence,% (95%CI^b^) |
| --- | --- | --- | --- | --- | --- |
| A40 | Streptococcal sepsis | 1350 | | 2.6(2.5, 2.7) | |
| A41 | Other sepsis | 1207 | | 2.3(2.2, 2.4) | |
| A50 | Congenital syphilis | 3015 | | 5.8(5.6, 6.0) | |
| B17 | Other acute viral hepatitis | 1112 | | 2.1(2.0, 2.3) | |
| B96 | Other specified bacterial agents as the cause of diseases classified to other chapters | 1470 | | 2.8(2.7, 3.0) | |
| C73 | Thyroid cancer | 482 | | 0.9(0.8, 1.0) | |
| C80 | Malignant neoplasm, without specification of site | 1880 | | 3.6(3.4, 3.8) | |
| D16 | Benign neoplasm of bone and articular cartilage | 2618 | | 5.0(4.8, 5.2) | |
| D50 | Iron deficiency anaemia | 2770 | | 5.3(5.1, 5.5) | |
| D61 | Other aplastic anaemias | 995 | | 1.9(1.8, 2.0) | |
| D62 | Acute posthaemorrhagic anaemia | 1573 | | 3.0(2.9, 3.2) | |
| D63 | Anaemia in chronic diseases classified elsewhere | 1402 | | 2.7(2.6, 2.8) | |
| D64 | Anaemias | 10136 | | 19.5(19.1, 19.8) | |
| D68 | Other coagulation defects | 1465 | | 2.8(2.7, 3.0) | |
| D69 | Purpura and other haemorrhagic conditions | 3227 | | 6.2(6.0, 6.4) | |
| D72 | Other disorders of white blood cells | 2783 | | 5.3(5.1, 5.5) | |
| E03 | Hypothyroidism | 44999 | | 86.4(86.1, 86.7) | |
| E04 | Nontoxic goitre | 3777 | | 7.2(7.0, 7.5) | |
| E05 | Hyperthyroidism] | 2371 | | 4.6(4.4, 4.7) | |
| E06 | Thyroiditis | 774 | | 1.5(1.4, 1.6) | |
| E10 | Type 1 diabetes mellitus | 10627 | | 20.4(20.0, 20.7) | |
| E11 | Diabetes mellitus | 5731 | | 11.0(10.7, 11.3) | |
| E20 | Hypoparathyroidism | 461 | | 0.9(0.8, 1.0) | |
| E55 | Vitamin D deficiency | 1295 | | 2.5(2.4, 2.6) | |
| E66 | Obesity | 2988 | | 5.7(5.5, 5.9) | |
| E67 | Other hyperalimentation | 3248 | | 6.2(6.0, 6.4) | |
| E78 | Disorders of lipoprotein metabolism | 23120 | | 44.4(43.9, 44.8) | |
| E83 | Disorders of mineral metabolism | 2182 | | 4.2(4.0, 4.4) | |
| E86 | Volume depletion | 1625 | | 3.1(3.0, 3.3) | |
| E87 | Disorders of fluid, electrolyte and acid-base balance | 12128 | | 23.3(22.9, 23.6) | |
| F09 | Unspecified organic or symptomatic mental disorder | 2168 | | 4.2(4.0, 4.3) | |
| F10 | Mental and behavioural disorders due to use of alcohol | 919 | | 1.8(1.7, 1.9) | |
| F17 | Mental and behavioural disorders due to use of tobacco | 1353 | | 2.6(2.5, 2.7) | |
| F19 | Mental and behavioural disorders due to multiple drug use and use of other psychoactive substances | 3180 | | 6.1(5.9, 6.3) | |
| F30 | Manic episode | 1840 | | 3.5(3.4, 3.7) | |
| F32 | Depressive episode | 10162 | | 19.5(19.2, 19.8) | |
| F41 | Other anxiety disorders | 3805 | | 7.3(7.1, 7.5) | |
| F43 | Reaction to severe stress, and adjustment disorders | 1210 | | 2.3(2.2, 2.5) | |
| G00 | Bacterial meningitis, not elsewhere classified | 2848 | | 5.5(5.3, 5.7) | |
| G31 | Degenerative diseases of nervous system | 1224 | | 2.3(2.2, 2.5) | |
| G41 | Status epilepticus | 1538 | | 3.0(2.8, 3.1) | |
| G43 | Migraine | 1896 | | 3.6(3.5, 3.8) | |
| G47 | Sleep disorders | 3738 | | 7.2(7.0, 7.4) | |
| G58 | Other mononeuropathies | 4393 | | 8.4(8.2, 8.7) | |
| G92 | Toxic encephalopathy | 999 | | 1.9(1.8, 2.0) | |
| G93 | Other disorders of brain | 5169 | | 9.9(9.7, 10.2) | |
| H35 | Other retinal disorders | 1661 | | 3.2(3.0, 3.3) | |
| H40 | Glaucoma | 1855 | | 3.6(3.4, 3.7) | |
| I10 | hypertension | 21951 | | 42.1(41.7, 42.5) | |
| I11 | Hypertensive heart disease | 1632 | | 3.1(3.0, 3.3) | |
| I12 | Hypertensive renal disease | 8108 | | 15.6(15.2, 15.9) | |
| I13 | Hypertensive heart and renal disease | 2023 | | 3.9(3.7, 4.0) | |
| I21 | Acute myocardial infarction | 1897 | | 3.6(3.5, 3.8) | |
| I25 | Ischemic heart disease | 12724 | | 24.4(24.0, 24.8) | |
| I27 | Other pulmonary heart diseases | 2889 | | 5.5(5.3, 5.7) | |
| I33 | Acute and subacute endocarditis | 2477 | | 4.8(4.6, 4.9) | |
| I42 | Cardiomyopathy | 1816 | | 3.5(3.3, 3.6) | |
| I45 | Other conduction disorders | 1291 | | 2.5(2.3, 2.6) | |
| I48 | Atrial fibrillation and flutter | 4839 | | 9.3(9.0, 9.5) | |
| I49 | Cardiac arrhythmias | 9002 | | 17.3(17.0, 17.6) | |
| I50 | Heart failure | 12107 | | 23.2(22.9, 23.6) | |
| I69 | Sequelae of cerebrovascular disease | 1555 | | 3.0(2.8, 3.1) | |
| I70 | Atherosclerosis | 1369 | | 2.6(2.5, 2.8) | |
| I79 | Disorders of arteries, arterioles and capillaries | 1404 | | 2.7(2.6, 2.8) | |
| I82 | Other venous embolism and thrombosis | 1155 | | 2.2(2.1, 2.3) | |
| I95 | Hypotension | 4578 | | 8.8(8.5, 9.0) | |
| J18 | Pneumonia | 2857 | | 5.5(5.3, 5.7) | |
| J39 | Other diseases of upper respiratory tract | 425 | | 0.8(0.7, 0.9) | |
| J44 | Chronic obstructive pulmonary disease | 4566 | | 8.8(8.5, 9.0) | |
| J45 | Asthma | 6313 | | 12.1(11.8, 12.4) | |
| J69 | Pneumonitis due to solids and liquids | 1268 | | 2.4(2.3, 2.6) | |
| J96 | Respiratory failure, not elsewhere classified | 1927 | | 3.7(3.5, 3.9) | |
| J99 | Respiratory disorders in diseases classified elsewhere | 2938 | | 5.6(5.4, 5.8) | |
| K21 | Gastro-oesophageal reflux disease | 6247 | | 12.0(11.7, 12.3) | |
| K23 | Disorders of oesophagus in diseases classified elsewhere | 8894 | | 17.1(16.7, 17.4) | |
| K29 | Gastritis and duodenitis | 1189 | | 2.3(2.2, 2.4) | |
| K31 | Diseases of stomach and duodenum | 1469 | | 2.8(2.7, 3.0) | |
| K56 | Paralytic ileus and intestinal obstruction without hernia | 1378 | | 2.6(2.5, 2.8) | |
| K57 | Diverticular disease of intestine | 1679 | | 3.2(3.1, 3.4) | |
| K59 | Other functional intestinal disorders | 1759 | | 3.4(3.2, 3.5) | |
| K63 | Diseases of intestine | 1159 | | 2.2(2.1, 2.4) | |
| K74 | Fibrosis and cirrhosis of liver | 2422 | | 4.6(4.5, 4.8) | |
| K76 | Liver disease | 1542 | | 3.0(2.8, 3.1) | |
| K77 | Liver disorders in diseases classified elsewhere | 783 | | 1.5(1.4, 1.6) | |
| K80 | Cholelithiasis | 1175 | | 2.3(2.1, 2.4) | |
| K86 | Other diseases of pancreas | 1166 | | 2.2(2.1, 2.4) | |
| K92 | Other diseases of digestive system | 1178 | | 2.3(2.1, 2.4) | |
| K93 | Disorders of other digestive organs in diseases classified elsewhere | 2661 | | 5.1(4.9, 5.3) | |
| L02 | Cutaneous abscess, furuncle and carbuncle | 1848 | | 3.5(3.4, 3.7) | |
| L03 | Cellulitis | 2078 | | 4.0(3.8, 4.2) | |
| M06 | Other rheumatoid arthritis | 1803 | | 3.5(3.3, 3.6) | |
| M10 | Gout | 3289 | | 6.3(6.1, 6.5) | |
| M12 | Other specific arthropathies | 1397 | | 2.7(2.5, 2.8) | |
| M19 | Other arthrosis | 1107 | | 2.1(2.0, 2.2) | |
| M53 | Other dorsopathies, not elsewhere classified | 2312 | | 4.4(4.3, 4.6) | |
| M54 | Dorsalgia | 1335 | | 2.6(2.4, 2.7) | |
| M70 | Soft tissue disorders related to use, overuse and pressure | 1932 | | 3.7(3.5, 3.9) | |
| M79 | Other soft tissue disorders, not elsewhere classified | 2554 | | 4.9(4.7, 5.1) | |
| M81 | Osteoporosis without pathological fracture | 1404 | | 2.7(2.6, 2.8) | |
| M93 | Other osteochondropathies | 4443 | | 8.5(8.3, 8.8) | |
| N01 | Rapidly progressive nephritic syndrome | 5208 | | 10.0(9.7, 10.3) | |
| N03 | Chronic nephritic syndrome | 6815 | | 13.1(12.8, 13.4) | |
| N17 | Acute renal failure | 3971 | | 7.6(7.4, 7.8) | |
| N18 | Chronic renal failure | 4827 | | 9.3(9.0, 9.5) | |
| N37 | Urethral disorders in diseases classified elsewhere | 4216 | | 8.1(7.9, 8.3) | |
| N39 | Disorders of urinary system | 2212 | | 4.2(4.1, 4.4) | |
| N40 | Hyperplasia of prostate | 2737 | | 5.3(5.1, 5.4) | |

^a^ICD-10: International Classification of Diseases codes, 10th Revision

^b^CI: confidence interval.

Table S3. Absolute prevalence differences of enrichment comorbidities in subgroups of patients with different thyroid disease types on the private dataset.

| ICD-10^a^ | Enrichment comorbidity | Absolute prevalence difference, value (95% CI^b^) | | |
| --- | --- | --- | --- | --- |
|  |  | TC-Hypothyroidism | TC^c^-Nontoxic goitre | TC-Hyperthyroidism |
| I10 | Hypertension | -24.9(-26.9, -22.9) | -25.9(-28.1, -23.7) | -16.0(-19.7, -12.3) |
| E03 | Hypothyroidism | 8.6(6.7, 10.5) | -85.8(-87.7, -83.9) | -13.4(-17.1, -9.7) |
| K76 | Liver disease | -21.2(-22.8, -19.6) | -10.4(-12.0, -8.8) | -12.6(-15.7, -9.5) |
| E11 | Diabetes mellitus | -15.0(-16.2, -13.8) | -13.4(-14.8, -12.0) | -9.3(-11.9, -6.7) |
| I70 | Atherosclerosis | -17.6(-18.6, -16.6) | -11.0(-12.1, -9.9) | -13.1(-15.8, -10.4) |
| E78 | Disorders of lipoprotein metabolism | -12.6(-13.9, -11.3) | -13.2(-14.6, -11.8) | -7.8(-10.3, -5.3) |
| I25 | Ischemic heart disease | -13.4(-14.3, -12.5) | -16.3(-17.5, -15.1) | -9.5(-11.8, -7.2) |
| K29 | Gastritis and duodenitis | -15.0(-15.9, -14.1) | -10.7(-11.7, -9.7) | -9.8(-12.1, -7.5) |
| N28 | Disorders of kidney and ureter | -13.2(-14.2, -12.2) | -7.7(-8.7, -6.7) | -5.4(-7.4, -3.4) |
| J15 | Bacterial pneumonia | -9.5(-10.2, -8.8) | -15.6(-16.6, -14.6) | -4.9(-6.6, -3.2) |
| I63 | Cerebral infarction | -10.4(-11.0, -9.8) | -8.6(-9.3, -7.9) | -4.7(-6.2, -3.2) |
| I11 | Hypertensive heart disease | -9.5(-10.2, -8.8) | -8.9(-9.8, -8.0) | -6.8(-8.7, -4.9) |
| J18 | Pneumonia | -6.1(-7.3, -4.9) | -5.0(-6.3, -3.7) |  |
| C77 | Secondary malignant neoplasm of lymph nodes | 31.5(28.6, 34.4) | 32.9(30.0, 35.8) | 36.7(33.7, 39.7) |
| K80 | Cholelithiasis | -8.3(-9.3, -7.3) | -6.9(-8.0, -5.8) | -5.5(-7.5, -3.5) |
| D64 | Anaemias | -5.3(-6.3, -4.3) | -9.3(-10.4, -8.2) | -3.8(-5.7, -1.9) |
| C34 | Malignant neoplasm of bronchus and lung | -8.5(-9.2, -7.8) | -5.7(-6.5, -4.9) |  |
| N40 | Hyperplasia of prostate | -8.9(-9.5, -8.3) | -5.2(-5.9, -4.5) | -4.5(-6.1, -2.9) |
| I50 | Heart failure | -4.8(-5.2, -4.4) | -12.6(-13.4, -11.8) | -8.9(-11.0, -6.8) |
| M81 | Osteoporosis without pathological fracture | -7.8(-8.4, -7.2) | -6.7(-7.4, -6.0) | -12.2(-14.6, -9.8) |
| E87 | Disorders of fluid, electrolyte and acid-base balance | -2.3(-3.5, -1.1) | -5.1(-6.4, -3.8) | -2.6(-4.7, -0.5) |
| C78 | Secondary malignant neoplasm of respiratory and digestive organs | -2.6(-3.8, -1.4) | -3.5(-4.8, -2.2) | 2.0(0.6, 3.4) |
| C73 | Malignant neoplasm of thyroid gland | 100.0(100.0, 100.0) | 100.0(100.0, 100.0) | 100.0(100.0, 100.0) |
| N18 | Chronic renal failure | -4.1(-4.6, -3.6) | -8.0(-8.7, -7.3) | -3.6(-5.0, -2.2) |
| E79 | Disorders of purine and pyrimidine metabolism | -2.4(-3.4, -1.4) | -4.2(-5.3, -3.1) | -1.9(-3.6, -0.2) |
| N20 | Calculus of kidney and ureter | -4.9(-5.7, -4.1) | -2.9(-3.7, -2.1) | -2.6(-4.2, -1.0) |
| I49 | Cardiac arrhythmias | -5.2(-5.7, -4.7) | -5.4(-6.1, -4.7) | -4.1(-5.6, -2.6) |
| C79 | Secondary malignant neoplasm | -3.2(-4.2, -2.2) | -2.7(-3.7, -1.7) |  |
| E55 | Vitamin D deficiency | -4.6(-5.1, -4.1) | -6.0(-6.7, -5.3) | -9.6(-11.8, -7.4) |
| M48 | Spondylopathies | -5.1(-5.6, -4.6) | -4.2(-4.8, -3.6) |  |
| E77 | Disorders of glycoprotein metabolism | -2.4(-3.1, -1.7) | -4.5(-5.4, -3.6) |  |
| I67 | Cerebrovascular diseases | -4.7(-5.2, -4.2) | -3.6(-4.1, -3.1) |  |
| I20 | Angina pectoris | -3.4(-3.8, -3.0) | -6.1(-6.7, -5.5) |  |
| E05 | Hyperthyroidism | -3.4(-4.2, -2.6) | -1.5(-2.3, -0.7) | -98.4(-99.1, -97.7) |
| J94 | Pleural conditions | -3.1(-3.6, -2.6) | -5.2(-5.9, -4.5) |  |
| J43 | Emphysema | -3.3(-4.1, -2.5) | -1.6(-2.4, -0.8) |  |
| N08 | Glomerular disorders | -3.6(-4.0, -3.2) | -5.2(-5.8, -4.6) |  |
| M51 | Intervertebral disc disorders | -4.0(-4.5, -3.5) | -4.0(-4.6, -3.4) |  |
| I79 | Disorders of arteries, arterioles and capillaries | -4.6(-5.0, -4.2) |  | -4.4(-5.9, -2.9) |
| B66 | Fluke infections | -3.5(-4.1, -2.9) | -2.0(-2.6, -1.4) | -3.7(-5.2, -2.2) |
| E27 | Disorders of adrenal gland | -2.5(-2.9, -2.1) | -4.9(-5.5, -4.3) |  |
| K82 | Diseases of gallbladder | -2.4(-3.2, -1.6) |  |  |
| G63 | Polyneuropathy diseases | -3.8(-4.2, -3.4) | -2.6(-3.0, -2.2) |  |
| D25 | Leiomyoma of uterus | -3.6(-4.2, -3.0) | -1.6(-2.2, -1.0) |  |
| I48 | Atrial fibrillation and flutter | -2.4(-2.7, -2.1) | -4.6(-5.2, -4.0) | -7.3(-9.2, -5.4) |
| D18 | Haemangioma and lymphangioma | -2.8(-3.5, -2.1) |  |  |
| N39 | Disorders of urinary system | -3.1(-3.4, -2.8) | -3.4(-3.9, -2.9) |  |
| N04 | Nephrotic syndrome | | -6.6(-7.2, -6.0) |  |
| J32 | Chronic sinusitis | -2.5(-3.1, -1.9) | -1.9(-2.6, -1.2) |  |
| I65 | Occlusion and stenosis of precerebral arteries | -3.3(-3.6, -3.0) | -2.9(-3.3, -2.5) |  |
| K31 | Diseases of stomach and duodenum | -3.7(-4.1, -3.3) |  |  |
| I69 | Sequelae of cerebrovascular disease | -2.4(-2.8, -2.0) | -3.4(-4.0, -2.8) |  |
| A49 | Bacterial infection | -3.0(-3.5, -2.5) |  |  |
| G47 | Sleep disorders | -3.0(-3.4, -2.6) | -2.4(-2.8, -2.0) | -4.1(-5.6, -2.6) |
| K63 | Diseases of intestine | -3.3(-3.6, -3.0) |  |  |
| C50 | Malignant neoplasm of breast | -2.5(-2.8, -2.2) | -2.2(-2.6, -1.8) |  |
| M10 | Gout | -1.8(-2.2, -1.4) | -2.9(-3.4, -2.4) |  |
| M50 | Cervical disc disorders | -2.9(-3.3, -2.5) |  |  |
| K74 | Fibrosis and cirrhosis of liver | | -3.1(-3.7, -2.5) |  |
| G31 | Degenerative diseases of nervous system | -2.6(-2.9, -2.3) |  |  |
| M35 | Systemic involvement of connective tissue | | -3.0(-3.5, -2.5) |  |
| J44 | Chronic obstructive pulmonary disease | -1.8(-2.0, -1.6) | -2.4(-2.8, -2.0) |  |
| E06 | Thyroiditis | 3.4(2.1, 4.7) | 2.7(1.4, 4.0) | 3.8(2.3, 5.3) |
| M80 | Osteoporosis with pathological fracture | -2.1(-2.4, -1.8) |  | -4.2(-5.7, -2.7) |
| C22 | Malignant neoplasm of liver and intrahepatic bile ducts | | -3.4(-3.9, -2.9) |  |
| C90 | Multiple myeloma and malignant plasma cell neoplasms | -2.3(-2.6, -2.0) |  | -3.2(-4.5, -1.9) |
| C11 | Malignant neoplasm of nasopharynx | | -2.6(-3.0, -2.2) |  |
| E83 | Disorders of mineral metabolism | 2.8(1.6, 4.0) | 2.2(1.0, 3.4) | 2.5(1.1, 3.9) |
| N83 | Noninflammatory disorders of ovary, fallopian tube and broad ligament | -1.9(-2.2, -1.6) |  |  |
| N60 | Benign mammary dysplasia | -1.1(-1.7, -0.5) |  |  |

^a^ICD-10: International Classification of Diseases codes, 10th Revision

^a^CI: confidence interval.

^c^TC: thyroid cancer.

Table S4. Absolute prevalence differences of enrichment comorbidities in subgroups of patients on the MIMIC-IV dataset.

| ICD-10^a^ | Enrichment comorbidity | Crude prevalence (95%CI^b^) | Absolute prevalence difference, value (95% CI) | | | | |
| --- | --- | --- | --- | --- | --- | --- | --- |
|  |  |  | Male-Female | TC^c^-BTD^d^ | TC-Hypothyroidism | TC-Nontoxic goitre | TC-Hyperthyroidism |
| E78 | Disorders of lipoprotein metabolism | 44.4(43.9, 44.8) | - | -15.5(-19.6, -11.4) | -42.5(-44.2, -40.8) | -44.8(-45.3, -44.3) | -37.1(-39.2, -35.0) |
| I25 | Ischemic heart disease | 24.4(24.0, 24.8) | 17.2(16.3, 18.1) | -16.9(-19.3, -14.5) | -18.7(-20.0, -17.4) | -25.2(-25.6, -24.8) | -17.6(-19.2, -16.0) |
| E87 | Disorders of fluid, electrolyte and acid-base balance | 23.3(22.9, 23.6) | - | -15.9(-18.3, -13.5) | -20.0(-21.4, -18.6) | -23.6(-24.0, -23.2) | -19.5(-21.2, -17.8) |
| I50 | Heart failure | 23.2(22.9, 23.6) | - | -19.9(-21.6, -18.2) | -18.0(-19.3, -16.7) | -23.7(-24.1, -23.3) | -19.6(-21.3, -17.9) |
| F32 | Depressive episode | 19.5(19.2, 19.8) | - | -11.1(-13.6, -8.6) | -16.4(-17.7, -15.1) | -20.0(-20.4, -19.6) | -16.6(-18.2, -15.0) |
| D64 | Anaemias | 19.5(19.1, 19.8) | - | -11.1(-13.6, -8.6) | -17.7(-19.0, -16.4) | -19.8(-20.2, -19.4) | -17.1(-18.7, -15.5) |
| I49 | Cardiac arrhythmias | 17.3(17.0, 17.6) | - | -9.1(-11.6, -6.6) | -14.9(-16.1, -13.7) | -17.4(-17.8, -17.0) | -18.8(-20.5, -17.1) |
| I12 | Hypertensive renal disease | 15.6(15.2, 15.9) | 7.7(7.0, 8.4) | -9.2(-11.4, -7.0) | -13.2(-14.3, -12.1) | -15.8(-16.1, -15.5) | -12.0(-13.4, -10.6) |
| N03 | Chronic nephritic syndrome | 13.1(12.8, 13.4) | 6.4(5.7, 7.1) | -6.0(-8.3, -3.7) | -10.2(-11.2, -9.2) | -13.4(-13.7, -13.1) | -10.1(-11.4, -8.8) |
| J45 | Asthma | 12.1(11.8, 12.4) | -7.0(-7.5, -6.5) | - | -11.8(-12.9, -10.7) | -12.0(-12.3, -11.7) | -13.7(-15.2, -12.2) |
| K21 | Gastro-oesophageal reflux disease | 12.0(11.7, 12.3) | - | -8.2(-10.0, -6.4) | -10.2(-11.2, -9.2) | -12.2(-12.5, -11.9) | -9.0(-10.2, -7.8) |
| E11 | Diabetes mellitus | 11.0(10.7, 11.3) | - | -8.8(-10.2, -7.4) | -10.4(-11.4, -9.4) | -10.8(-11.1, -10.5) | -9.7(-11.0, -8.4) |
| N01 | Rapidly progressive nephritic syndrome | 10.0(9.7, 10.3) | - | -7.0(-8.6, -5.4) | -8.4(-9.3, -7.5) | -10.3(-10.6, -10.0) | -7.9(-9.0, -6.8) |
| G93 | Other disorders of brain | 9.9(9.7, 10.2) | - | -4.0(-6.1, -1.9) | -10.9(-12.0, -9.8) | -10.0(-10.3, -9.7) | -9.8(-11.1, -8.5) |
| I48 | Atrial fibrillation and flutter | 9.3(9.0, 9.5) | 5.4(4.8, 6.0) | -8.4(-9.3, -7.5) | -7.2(-8.1, -6.3) | -9.3(-9.6, -9.0) | -9.8(-11.1, -8.5) |
| N18 | Chronic renal failure | 9.3(9.0, 9.5) | 6.5(5.9, 7.1) | -8.5(-9.3, -7.7) | -6.9(-7.8, -6.0) | -9.4(-9.7, -9.1) | -7.1(-8.2, -6.0) |
| I95 | Hypotension | 8.8(8.5, 9.0) | - | -5.5(-7.1, -3.9) | -8.3(-9.2, -7.4) | -8.8(-9.1, -8.5) | -8.5(-9.7, -7.3) |
| J44 | Chronic obstructive pulmonary disease | 8.8(8.5, 9.0) | - | -6.7(-8.0, -5.4) | -7.2(-8.1, -6.3) | -8.9(-9.2, -8.6) | -8.3(-9.5, -7.1) |
| M93 | Other osteochondropathies | 8.5(8.3, 8.8) | -6.5(-6.9, -6.1) | -3.8(-5.7, -1.9) | -9.1(-10.1, -8.1) | -8.6(-8.9, -8.3) | -7.4(-8.5, -6.3) |
| G58 | Other mononeuropathies | 8.4(8.2, 8.7) | -3.7(-4.2, -3.2) | -3.9(-5.8, -2.0) | -8.7(-9.7, -7.7) | -8.5(-8.8, -8.2) | -8.9(-10.1, -7.7) |
| N37 | Urethral disorders in diseases classified elsewhere | 8.1(7.9, 8.3) | -3.2(-3.7, -2.7) | -7.4(-8.2, -6.6) | -6.9(-7.8, -6.0) | -8.3(-8.6, -8.0) | -6.9(-8.0, -5.8) |
| N17 | Acute renal failure | 7.6(7.4, 7.8) | 3.7(3.1, 4.3) | -7.5(-8.0, -7.0) | -5.6(-6.4, -4.8) | -7.8(-8.0, -7.6) | -5.7(-6.7, -4.7) |
| F41 | Other anxiety disorders | 7.3(7.1, 7.5) | - | -5.3(-6.6, -4.0) | -6.2(-7.0, -5.4) | -7.4(-7.6, -7.2) | -5.5(-6.5, -4.5) |
| G47 | Sleep disorders | 7.2(7.0, 7.4) | 3.2(2.7, 3.7) | -4.5(-6.0, -3.0) | -6.3(-7.1, -5.5) | -7.3(-7.5, -7.1) | -4.8(-5.7, -3.9) |
| M10 | Gout | 6.3(6.1, 6.5) | 7.8(7.2, 8.4) | -2.6(-4.3, -0.9) | -5.0(-5.7, -4.3) | -6.6(-6.8, -6.4) | -3.9(-4.7, -3.1) |
| D69 | Purpura and other haemorrhagic conditions | 6.2(6.0, 6.4) | 3.5(3.0, 4.0) | -3.9(-5.3, -2.5) | -5.5(-6.3, -4.7) | -6.3(-6.5, -6.1) | -5.0(-5.9, -4.1) |
| A50 | Congenital syphilis | 5.8(5.6, 6.0) | - | -4.8(-5.7, -3.9) | -5.1(-5.8, -4.4) | -5.9(-6.1, -5.7) | -4.5(-5.4, -3.6) |
| G00 | Bacterial meningitis, not elsewhere classified | 5.5(5.3, 5.7) | 2.6(2.1, 3.1) | 3.3(0.8, 5.8) | -5.4(-6.2, -4.6) | -5.5(-5.7, -5.3) | -4.4(-5.3, -3.5) |
| I27 | Other pulmonary heart diseases | 5.5(5.3, 5.7) | - | -3.7(-4.9, -2.5) | -5.0(-5.7, -4.3) | -5.5(-5.7, -5.3) | -5.8(-6.8, -4.8) |
| J18 | Pneumonia | 5.5(5.3, 5.7) | - | -3.6(-4.8, -2.4) | -4.5(-5.2, -3.8) | -5.5(-5.7, -5.3) | -4.5(-5.4, -3.6) |
| D50 | Iron deficiency anaemia | 5.3(5.1, 5.5) | - | -3.2(-4.5, -1.9) | -5.6(-6.4, -4.8) | -5.3(-5.5, -5.1) | -5.2(-6.1, -4.3) |
| D72 | Other disorders of white blood cells | 5.3(5.1, 5.5) | - | -3.3(-4.6, -2.0) | -5.6(-6.4, -4.8) | -5.3(-5.5, -5.1) | -4.9(-5.8, -4.0) |
| N40 | Hyperplasia of prostate | 5.3(5.1, 5.4) | 18.6(18.0, 19.2) | -2.0(-3.6, -0.4) | -5.2(-6.0, -4.4) | -5.3(-5.5, -5.1) | -4.1(-4.9, -3.3) |
| K93 | Disorders of other digestive organs in diseases classified elsewhere | 5.1(4.9, 5.3) | - | -2.0(-3.6, -0.4) | -4.9(-5.6, -4.2) | -5.2(-5.4, -5.0) | -4.2(-5.1, -3.3) |
| D16 | Benign neoplasm of bone and articular cartilage | 5.0(4.8, 5.2) | -2.7(-3.1, -2.3) | -3.9(-4.9, -2.9) | -5.4(-6.2, -4.6) | -5.0(-5.2, -4.8) | -4.0(-4.8, -3.2) |
| M79 | Other soft tissue disorders, not elsewhere classified | 4.9(4.7, 5.1) | - | -2.4(-3.8, -1.0) | -3.9(-4.6, -3.2) | -4.9(-5.1, -4.7) | -4.6(-5.5, -3.7) |
| I33 | Acute and subacute endocarditis | 4.8(4.6, 4.9) | - | -2.9(-4.1, -1.7) | -4.2(-4.9, -3.5) | -4.8(-5.0, -4.6) | -4.7(-5.6, -3.8) |
| K74 | Fibrosis and cirrhosis of liver | 4.6(4.5, 4.8) | 2.4(2.0, 2.8) | - | -4.4(-5.1, -3.7) | -4.8(-5.0, -4.6) | -3.3(-4.1, -2.5) |
| N39 | Disorders of urinary system | 4.2(4.1, 4.4) | -1.7(-2.0, -1.4) | -3.9(-4.5, -3.3) | -3.3(-3.9, -2.7) | -4.3(-4.5, -4.1) | -3.1(-3.8, -2.4) |
| E83 | Disorders of mineral metabolism | 4.2(4.0, 4.4) | - | 3.5(1.1, 5.9) | -4.2(-4.9, -3.5) | -4.1(-4.3, -3.9) | -4.5(-5.4, -3.6) |
| F09 | Unspecified organic or symptomatic mental disorder | 4.2(4.0, 4.3) | - | -3.4(-4.2, -2.6) | -3.8(-4.4, -3.2) | -4.3(-4.5, -4.1) | -4.0(-4.8, -3.2) |
| L03 | Cellulitis | 4.0(3.8, 4.2) | - | -3.2(-4.0, -2.4) | -3.3(-3.9, -2.7) | -4.2(-4.4, -4.0) | -2.7(-3.4, -2.0) |
| I13 | Hypertensive heart and renal disease | 3.9(3.7, 4.0) | 2.8(2.4, 3.2) | -3.7(-4.1, -3.3) | -2.5(-3.0, -2.0) | -4.0(-4.2, -3.8) | -3.5(-4.3, -2.7) |
| J96 | Respiratory failure, not elsewhere classified | 3.7(3.5, 3.9) | - | -2.9(-3.7, -2.1) | -3.2(-3.8, -2.6) | -3.7(-3.9, -3.5) | -2.8(-3.5, -2.1) |
| M70 | Soft tissue disorders related to use, overuse and pressure | 3.7(3.5, 3.9) | -2.2(-2.5, -1.9) | - | -3.9(-4.6, -3.2) | -3.7(-3.9, -3.5) | -4.0(-4.8, -3.2) |
| G43 | Migraine | 3.6(3.5, 3.8) | -3.7(-4.0, -3.4) | -2.2(-3.3, -1.1) | -4.3(-5.0, -3.6) | -3.6(-3.8, -3.4) | -5.0(-5.9, -4.1) |
| I21 | Acute myocardial infarction | 3.6(3.5, 3.8) | - | -3.1(-3.8, -2.4) | -2.5(-3.0, -2.0) | -3.8(-4.0, -3.6) |  |
| C80 | Malignant neoplasm, without specification of site | 3.6(3.4, 3.8) | - | 18.8(15.1, 22.5) | -2.5(-3.0, -2.0) | -3.5(-3.7, -3.3) | -2.8(-3.5, -2.1) |
| H40 | Glaucoma | 3.6(3.4, 3.7) | - | -1.9(-3.1, -0.7) | -4.2(-4.9, -3.5) | -3.5(-3.7, -3.3) | -3.4(-4.2, -2.6) |
| F30 | Manic episode | 3.5(3.4, 3.7) | - | -1.6(-2.8, -0.4) | -2.7(-3.2, -2.2) | -3.6(-3.8, -3.4) | -3.4(-4.2, -2.6) |
| L02 | Cutaneous abscess, furuncle and carbuncle | 3.5(3.4, 3.7) | 1.7(1.3, 2.1) | -2.6(-3.5, -1.7) | -2.5(-3.0, -2.0) | -3.7(-3.9, -3.5) |  |
| I42 | Cardiomyopathy | 3.5(3.3, 3.6) | 3.0(2.6, 3.4) | -2.0(-3.1, -0.9) | -2.3(-2.8, -1.8) | -3.5(-3.7, -3.3) | -4.0(-4.8, -3.2) |
| M06 | Other rheumatoid arthritis | 3.5(3.3, 3.6) | -2.6(-2.9, -2.3) | -2.0(-3.1, -0.9) | -2.5(-3.0, -2.0) | -3.5(-3.7, -3.3) | -3.3(-4.1, -2.5) |
| K59 | Other functional intestinal disorders | 3.4(3.2, 3.5) | - | -3.4(-3.6, -3.2) | -3.2(-3.8, -2.6) | -3.4(-3.6, -3.2) | -2.8(-3.5, -2.1) |
| K57 | Diverticular disease of intestine | 3.2(3.1, 3.4) | - | -2.6(-3.3, -1.9) | -3.5(-4.1, -2.9) | -3.2(-3.4, -3.0) | -2.9(-3.6, -2.2) |
| H35 | Other retinal disorders | 3.2(3.0, 3.3) | - | -2.6(-3.3, -1.9) | -3.2(-3.8, -2.6) | -3.2(-3.4, -3.0) | -3.0(-3.7, -2.3) |
| E86 | Volume depletion | 3.1(3.0, 3.3) | - | -2.9(-3.3, -2.5) | -2.7(-3.2, -2.2) | -3.1(-3.3, -2.9) | -2.8(-3.5, -2.1) |
| I11 | Hypertensive heart disease | 3.1(3.0, 3.3) | - | -3.0(-3.4, -2.6) | -2.9(-3.5, -2.3) | -3.1(-3.3, -2.9) | -2.7(-3.4, -2.0) |
| D62 | Acute posthaemorrhagic anaemia | 3.0(2.9, 3.2) | - | -2.2(-3.0, -1.4) | -2.5(-3.0, -2.0) | -3.0(-3.2, -2.8) | -2.8(-3.5, -2.1) |
| G41 | Status epilepticus | 3.0(2.8, 3.1) | - | -2.2(-3.0, -1.4) | -2.9(-3.5, -2.3) | -3.1(-3.3, -2.9) |  |
| I69 | Sequelae of cerebrovascular disease | 3.0(2.8, 3.1) | - | -2.2(-3.0, -1.4) | -3.7(-4.3, -3.1) | -2.9(-3.1, -2.7) | -4.0(-4.8, -3.2) |
| K76 | Liver disease | 3.0(2.8, 3.1) | - | -1.8(-2.8, -0.8) | -3.1(-3.7, -2.5) | -3.0(-3.2, -2.8) |  |
| B96 | Other specified bacterial agents as the cause of diseases classified to other chapters | 2.8(2.7, 3.0) | - | -2.6(-3.0, -2.2) | -2.3(-2.8, -1.8) | -2.8(-3.0, -2.6) | -2.5(-3.2, -1.8) |
| D68 | Other coagulation defects | 2.8(2.7, 3.0) | - | -1.6(-2.6, -0.6) |  | -2.9(-3.1, -2.7) | -2.4(-3.1, -1.7) |
| K31 | Diseases of stomach and duodenum | 2.8(2.7, 3.0) | - | -2.2(-2.9, -1.5) |  | -2.8(-3.0, -2.6) | -4.1(-4.9, -3.3) |
| D63 | Anaemia in chronic diseases classified elsewhere | 2.7(2.6, 2.8) | 1.8(1.5, 2.1) | -2.3(-2.9, -1.7) |  | -2.7(-2.9, -2.5) |  |
| M81 | Osteoporosis without pathological fracture | 2.7(2.6, 2.8) | -2.6(-2.8, -2.4) | -2.1(-2.8, -1.4) | -2.8(-3.4, -2.2) | -2.6(-2.7, -2.5) | -2.4(-3.1, -1.7) |
| I70 | Atherosclerosis | 2.6(2.5, 2.8) | - | -2.2(-2.8, -1.6) |  | -2.7(-2.9, -2.5) |  |
| K56 | Paralytic ileus and intestinal obstruction without hernia | 2.6(2.5, 2.8) | - | -2.1(-2.8, -1.4) |  | -2.7(-2.9, -2.5) |  |
| A40 | Streptococcal sepsis | 2.6(2.5, 2.7) | - | -2.4(-2.8, -2.0) |  | -2.7(-2.9, -2.5) |  |
| F17 | Mental and behavioural disorders due to use of tobacco | 2.6(2.5, 2.7) | - | -2.0(-2.7, -1.3) | -2.8(-3.4, -2.2) | -2.5(-2.6, -2.4) | -3.0(-3.7, -2.3) |
| M54 | Dorsalgia | 2.6(2.4, 2.7) | - | -1.8(-2.6, -1.0) | -3.2(-3.8, -2.6) | -2.4(-2.5, -2.3) | -2.5(-3.2, -1.8) |
| I45 | Other conduction disorders | 2.5(2.3, 2.6) | 1.3(1.0, 1.6) | -2.3(-2.7, -1.9) |  | -2.5(-2.6, -2.4) |  |
| J69 | Pneumonitis due to solids and liquids | 2.4(2.3, 2.6) | 2.2(1.9, 2.5) | - | -2.0(-2.5, -1.5) | -2.4(-2.5, -2.3) | -2.5(-3.2, -1.8) |
| F43 | Reaction to severe stress, and adjustment disorders | 2.3(2.2, 2.5) | -1.2(-1.5, -0.9) | -1.5(-2.3, -0.7) |  | -2.4(-2.5, -2.3) |  |
| G31 | Degenerative diseases of nervous system | 2.3(2.2, 2.5) | - | -2.2(-2.6, -1.8) |  | -2.4(-2.5, -2.3) | -2.3(-2.9, -1.7) |
| A41 | Other sepsis | 2.3(2.2, 2.4) | - | -2.3(-2.4, -2.2) |  | -2.4(-2.5, -2.3) |  |
| K80 | Cholelithiasis | 2.3(2.1, 2.4) | - | -1.3(-2.2, -0.4) |  | -2.3(-2.4, -2.2) |  |
| K92 | Other diseases of digestive system | 2.3(2.1, 2.4) | - | -1.5(-2.3, -0.7) |  | -2.3(-2.4, -2.2) |  |
| K86 | Other diseases of pancreas | 2.2(2.1, 2.4) | - | -1.9(-2.5, -1.3) |  | -2.3(-2.4, -2.2) |  |
| I82 | Other venous embolism and thrombosis | 2.2(2.1, 2.3) | - | -1.2(-2.1, -0.3) | -2.5(-3.0, -2.0) | -2.2(-2.3, -2.1) |  |
| B17 | Other acute viral hepatitis | 2.1(2.0, 2.3) | 1.5(1.2, 1.8) | - | -2.3(-2.8, -1.8) | -2.1(-2.2, -2.0) | -2.7(-3.4, -2.0) |
| M19 | Other arthrosis | 2.1(2.0, 2.2) | -1.0(-1.2, -0.8) | -1.9(-2.3, -1.5) | -2.8(-3.4, -2.2) | -2.0(-2.1, -1.9) |  |
| G92 | Toxic encephalopathy | 1.9(1.8, 2.0) | - | -1.7(-2.1, -1.3) |  | -1.9(-2.0, -1.8) |  |
| F10 | Mental and behavioural disorders due to use of alcohol | 1.8(1.7, 1.9) | 1.7(1.4, 2.0) | -1.2(-1.9, -0.5) |  | -1.8(-1.9, -1.7) |  |
| K77 | Liver disorders in diseases classified elsewhere | 1.5(1.4, 1.6) | 1.3(1.0, 1.6) | - |  | -1.6(-1.7, -1.5) |  |
| E20 | Hypoparathyroidism | 0.9(0.8, 1.0) | -0.4(-0.6, -0.2) | 4.6(2.6, 6.6) |  |  |  |
| J39 | Other diseases of upper respiratory tract | 0.8(0.7, 0.9) | - | 3.1(1.4, 4.8) |  |  |  |

^a^ICD-10: International Classification of Diseases codes, 10th Revision

^a^CI: confidence interval.

^c^TC: thyroid cancer.

^d^BTD: benign thyroid disease.


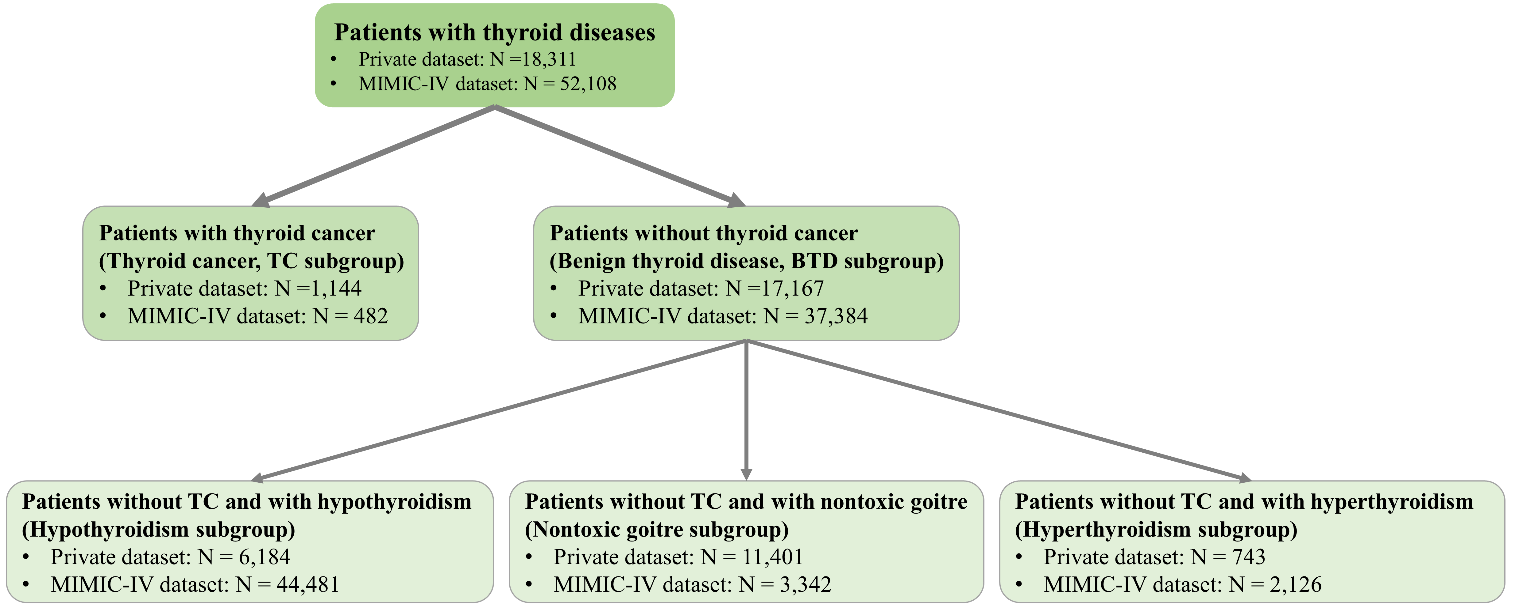


Figure S1. Patient subgroups categorized by thyroid disease types.


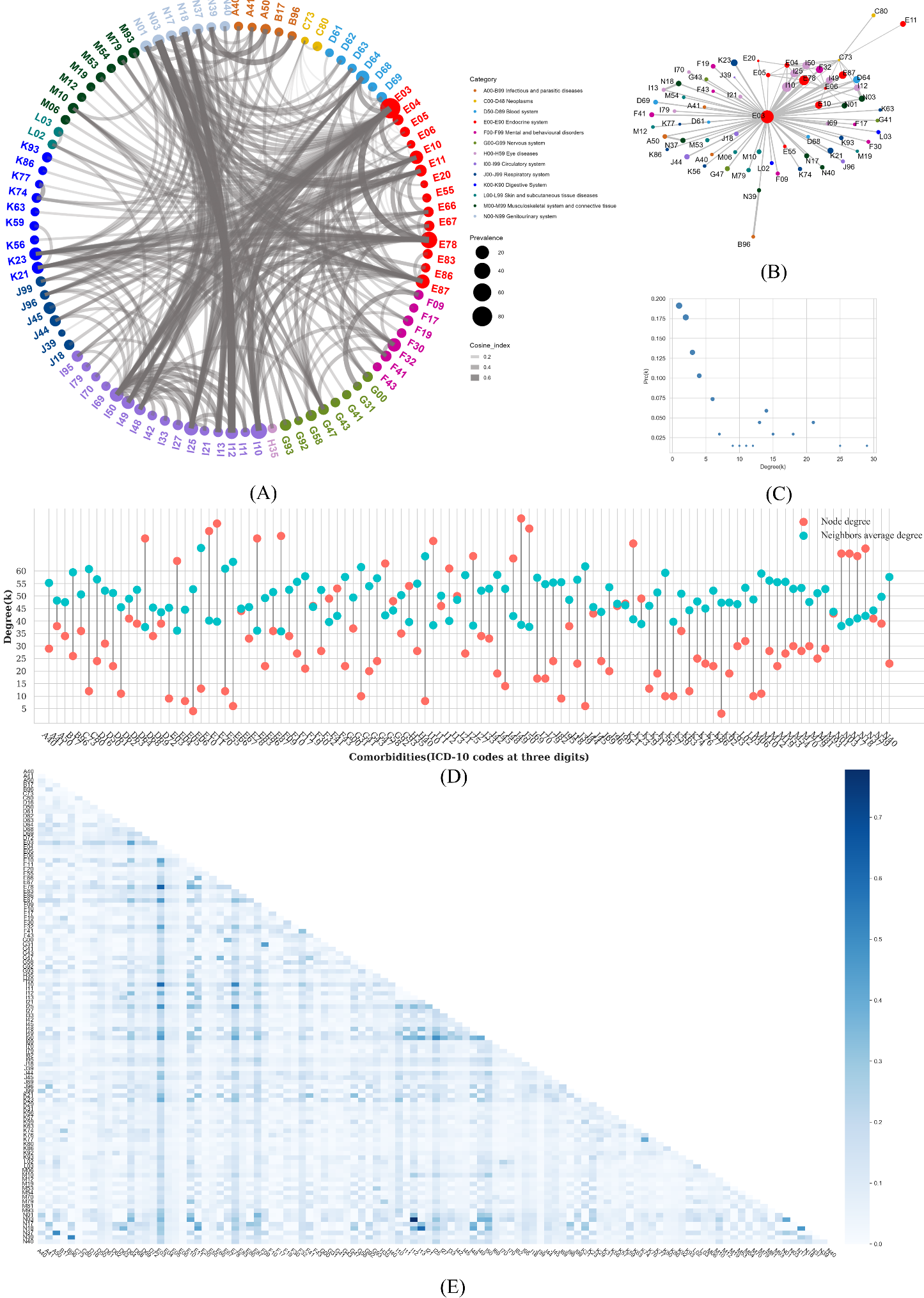


Figure S2. Phenotypic comorbidity network (PCN) in thyroid disease (TD) patients on the MIMIC-IV datasets. (A) The PCN of TD patients. Nodes represent comorbidities, and the node size was proportional to the comorbidity prevalence in TD patients and its color identified the disease category. Link weights were proportional to the magnitudes of the cosine index which were larger than 0. (B) The 100 edges in the PCN, where cosine index values were ≥0.20. (C) Cumulative degree (k) distribution of the PCN with 100 edges of (B). (D) The degree distribution of the node and its neighbors. Here, the connected nodes (degree >0) in the PCN were shown. (E) Correlations measured by cosine index between diseases. The disease codes were clarified in Table S1.


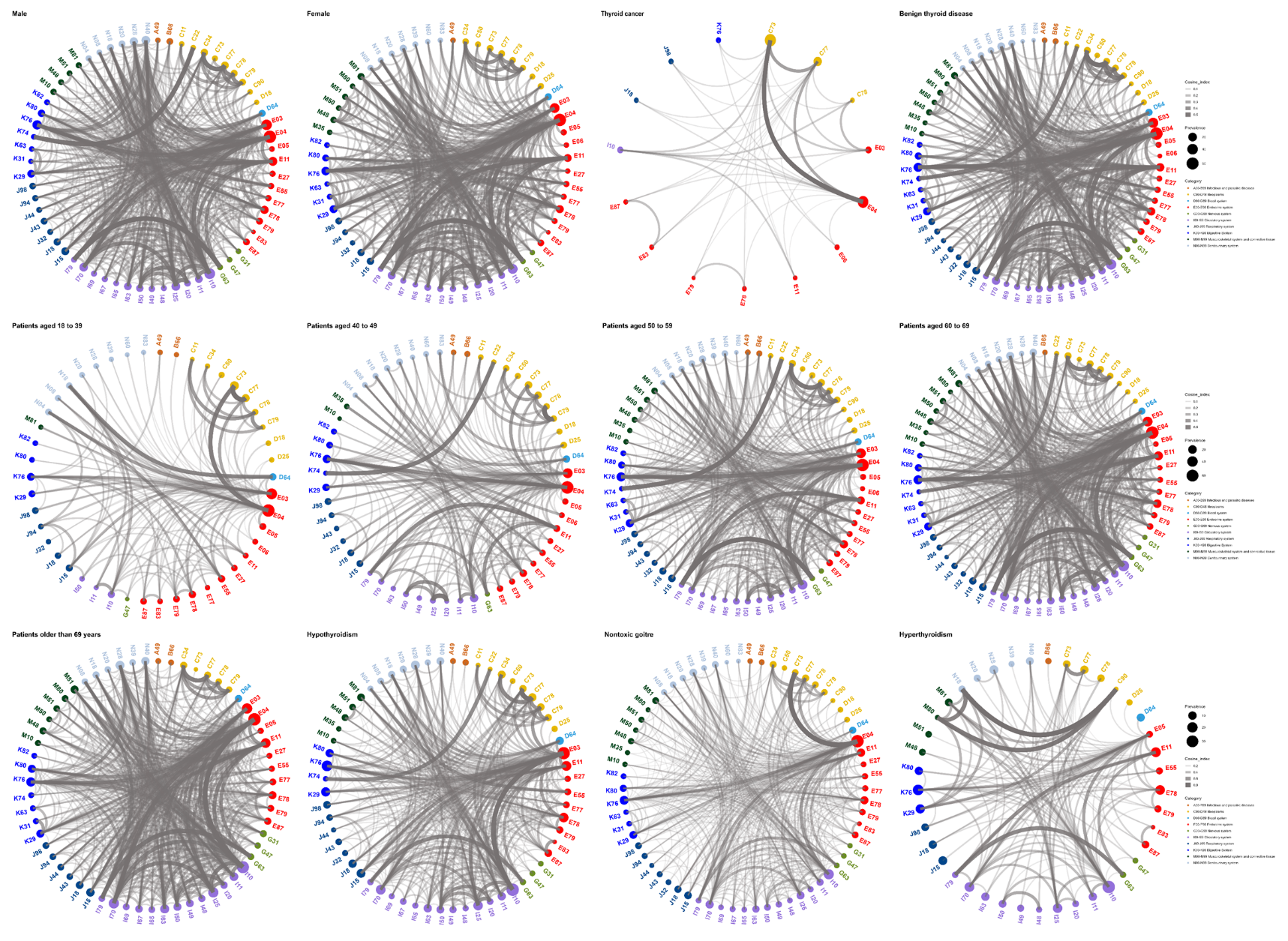


Figure S3. Phenotypic comorbidity network of thyroid disease patients in different subgroups on the private dataset.


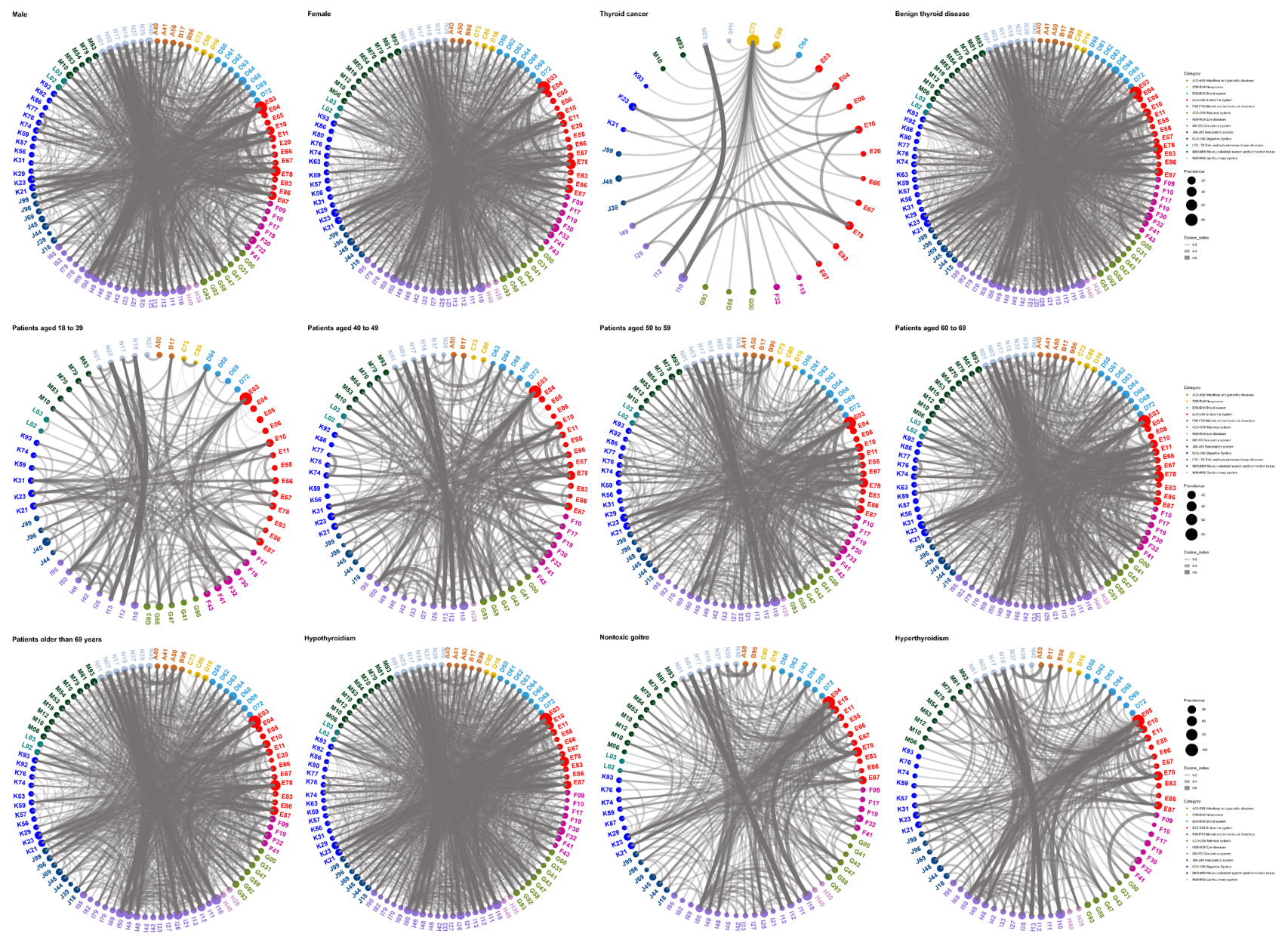


Figure S4. Phenotypic comorbidity network of thyroid disease patients in different subgroups on the MIMIC-IV dataset.


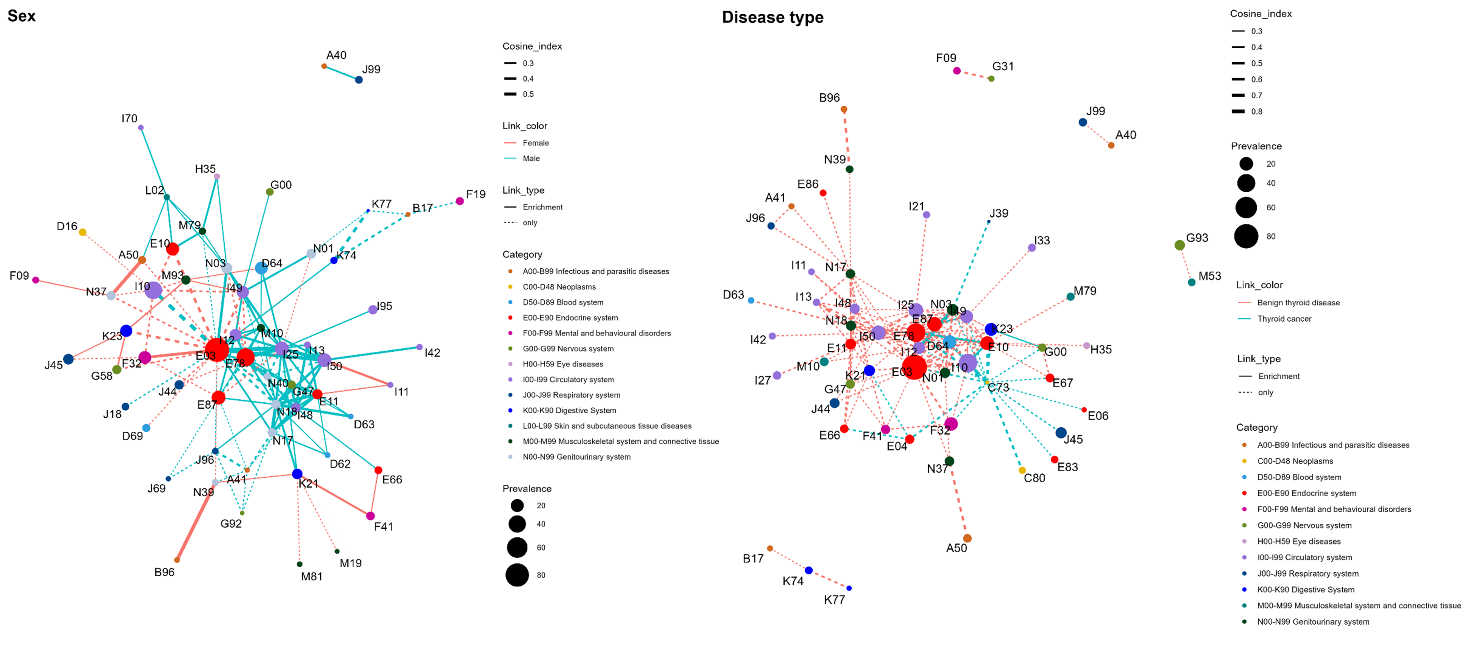


Figure S5. Abundant connections by sex and disease type in patients with thyroid disease on the MIMIC-IV dataset. Except for those common to both subgroups (link weight difference, i.e., cosine index <0.05), disease pairs were identified as abundant edges in one subgroup, including enrichment (solid lines) or only occurrence (dotted lines).
